# Efficacy and Safety of Inhaled Treprostinil in Idiopathic Pulmonary Fibrosis: A Systematic Review and Meta-Analysis

**DOI:** 10.3390/arm94050073

**Published:** 2026-10-08

**Authors:** Jyoti Bajpai, Akshyaya Pradhan, Gaurav Manek, Shivani Maurya, Surya Kant, Marco Alfonso Perrone

**Affiliations:** 1 Department of Respiratory Medicine, King George’s Medical University, Lucknow 226003, Uttar Pradesh, India; shivanimaurya88088@gmail.com (S.M.); skantpulmed@gmail.com (S.K.); 2 Department of Cardiology, King George’s Medical University, Lucknow 226003, Uttar Pradesh, India; akshyaya33@gmail.com; 3 Pulmonary and Critical Care Medicine, MedStar Medical Group, Clinton, MD 20735, USA; gaurav.manek10@gmail.com; 4 Department of Cardiology, University of Rome Tor Vergata, 00133 Rome, Italy; marco.perrone@uniroma2.it

**Keywords:** idiopathic pulmonary fibrosis, inhaled treprostinil, TETON, INCREASE, pulmonary hypertension, forced vital capacity, meta-analysis

## Abstract

**Highlights:**

**What are the main findings?**

- Inhaled treprostinil can slow the decline in lung function in IPF, independent of pulmonary hypertension.
- It also delays clinical worsening in IPF, suggesting a potential disease-modifying role beyond its established pulmonary vascular effects.

**What are the implications of the main findings?**

- Inhaled treprostinil is an inhaled prostacyclin analog that targets the pulmonary vascular component of fibrotic lung disease and has potential antiproliferative and anti-remodelling effects.

**Abstract:**

Background: Idiopathic pulmonary fibrosis (IPF) is a progressive fibrotic interstitial lung disease with high morbidity and mortality. Inhaled treprostinil showed pulmonary vascular and lung-function signals in pulmonary hypertension associated with Interstitial lung disease (PH-ILD) and was subsequently tested in IPF trials. Objectives: This systematic review and meta-analysis assessed the efficacy and safety of inhaled treprostinil in adults with IPF. Methods: PubMed, Google Scholar and the Cochrane Library/CENTRAL were searched for records published from January 2000 to 2026. A random-effects meta-analysis used the DerSimonian–Laird tau squared and inverse-variance weighting methods. Continuous outcomes were pooled as generic inverse-variance effects from reported estimates and 95% confidence intervals. Hazard ratios were pooled on the log scale. Safety outcomes were pooled as risk ratios from event counts. Results: The searches identified 1552 records. After deduplication, 379 records were screened and 85 full-text reports were assessed. Eleven reports were included in the systematic review. TETON-1 and TETON-2 were the two direct-IPF randomized trials within the coordinated TETON program eligible for primary meta-analysis. Inhaled treprostinil improved absolute forced vital capacity (FVC) change at 52 weeks compared with placebo (MD 111.28 mL, 95% CI 77.61 to 144.95, *p* < 0.001; I squared 8.5%). Clinical worsening was reduced (HR 0.69, 95% CI 0.57 to 0.84, *p* < 0.001; I squared 0.0%). Acute exacerbation was lower in pooled analysis (HR 0.52, 95% CI 0.30 to 0.91, *p* = 0.022), while all-cause mortality did not differ significantly (HR 0.70, 95% CI 0.47 to 1.05, *p* = 0.086). Adverse-event discontinuation, cough and headache were more frequent with treprostinil. Conclusions: In direct IPF randomized evidence, inhaled treprostinil preserved lung function and reduced clinical worsening at 52 weeks.

## 1. Introduction

Idiopathic pulmonary fibrosis is defined by progressive fibrotic lung injury and a usual interstitial pneumonia pattern after exclusion of known causes of interstitial lung disease [1,2,3]. The clinical course is variable, but many patients develop persistent decline in forced vital capacity, worsening gas exchange, respiratory hospitalization and death [4,5,6]. Acute exacerbation and forced vital capacity (FVC) decline are closely linked to later mortality, which makes preservation of lung function a clinically meaningful endpoint in IPF trials [7,8,9].

Pirfenidone and nintedanib have changed IPF management by slowing FVC decline in placebo-controlled trials [10,11,12]. However, treatment gaps are still clear in practice. Many patients progress despite background antifibrotic therapy, and the real-world uptake of therapy remains incomplete too [13,14]. Pulmonary hypertension also complicates advanced fibrotic lung disease and is associated with worse exercise capacity, higher symptom burden, and poor prognosis [15,16,17,18]. Prior trials of pulmonary vasodilator therapy in fibrotic interstitial lung disease were not uniformly positive, with safety concerns or limited efficacy in some settings [19,20].

Treprostinil is a prostacyclin analog delivered by inhalation for pulmonary vascular disease. Its inhaled route provides local pulmonary delivery and reduces systemic exposure compared with parenteral prostacyclin therapy [21]. Preclinical data show that treprostinil and prostacyclin receptor agonism can reduce fibroblast proliferation, extracellular matrix deposition, YAP/TAZ signaling, and experimental lung fibrosis [22,23,24,25]. These findings provided a mechanistic basis for testing inhaled treprostinil beyond pulmonary vasodilation.

The INCREASE trial tested inhaled treprostinil in pulmonary hypertension due to interstitial lung disease and showed improvement in 6 min walk distance, NT-proBNP and clinical worsening [26]. A post hoc analysis then found a favorable FVC signal, especially in the IPF subgroup [27]. The design of the TETON program was intended to test inhaled treprostinil directly in IPF using 52-week FVC change as the primary endpoint [28]. TETON-1 and TETON-2 later provided two separately randomized phase 3 placebo-controlled datasets within the coordinated TETON program in direct IPF populations [29,30].

This systematic review and meta-analysis evaluates inhaled treprostinil for efficacy and safety in IPF. The primary quantitative analysis was restricted to TETON-1 and TETON-2 to avoid double-counting and to preserve a direct-IPF evidence base. The INCREASE study, its post hoc analyses, open-label extension data and Japanese PH-ILD studies were assessed as secondary, sensitivity or narrative evidence according to design and population.

## 2. Methods

### 2.1. Reporting Framework and Eligibility Criteria

The review was structured according to PRISMA 2020 [31]. Eligible studies included adults with IPF or IPF represented within a pulmonary hypertension associated with Interstitial lung disease (PH-ILD) cohort, exposure to inhaled treprostinil and extractable efficacy or safety outcomes. Placebo-controlled direct IPF randomized trials were eligible for primary meta-analysis. PH-ILD randomized evidence, post hoc analyses, open-label extensions and single-arm studies were retained for secondary or narrative synthesis when they contained relevant IPF or PH-ILD data.

The review protocol, including eligibility criteria, outcomes and the planned analytical approach, was developed and finalized before study screening commenced. The protocol was not prospectively registered; it was subsequently registered retrospectively in PROSPERO (registration number: CRD420261497663). Retrospective registration is acknowledged as a methodological limitation.

The primary outcome was between-group change in absolute FVC in milliliters at week 52. Secondary efficacy outcomes included percentage predicted FVC, King’s Brief ILD (K-BILD) total score, percentage predicted diffusing capacity of lungs for carbon monoxide (DLCO), clinical worsening, acute exacerbation of IPF, mortality, 6 min walk distance and NT-pro-BNP where relevant. Safety outcomes included any adverse event, serious adverse events, treatment discontinuation due to adverse events, death and common adverse events.

### 2.2. Search Strategy and Study Selection

A structured search was conducted in PubMed, Google Scholar and the Cochrane Library/CENTRAL for records from January 2000 to 2026. The main search combined terms for treprostinil, inhaled treprostinil and Tyvaso with terms for idiopathic pulmonary fibrosis, interstitial lung disease, pulmonary fibrosis, pulmonary hypertension and PH-ILD. Focused searches for IPF, PH-ILD, TETON and INCREASE were also performed to verify retrieval of known eligible studies. Embase was not searched because database access was unavailable. The final search was run on 3 July 2026. TETON-2 and TETON-1 had been published online ahead of issue on 11 March 2026 and 18 May 2026, respectively, and were therefore available before the final search; both subsequently appeared in the 9 July 2026 issue [29,30]. Google Scholar was used as a broad supplementary identification source within the search strategy. Complete database-specific search strings, Boolean operators, field restrictions and focused verification searches are reproduced in Supplementary Table S1 and Supplementary Search S1A. Focused verification searches were not added to the PRISMA identification count.

The principal search sources identified 1552 records: 449 from PubMed, 913 from Google Scholar and 190 from the Cochrane Library/CENTRAL. After deduplication, 379 records were screened by title and abstract. Eighty-five full-text reports were assessed and 11 reports were retained for the final review.

Title and abstract screening and full-text eligibility assessment were performed independently by the first and fourth authors. Disagreements were recorded and resolved through adjudication meetings involving all authors until consensus was reached.

### 2.3. Data Extraction and Risk of Bias Assessment

Study characteristics, population details, intervention and comparator details, outcome definitions, effect estimates, confidence intervals and adverse-event counts were extracted into structured tables. The review separated independent patient-level datasets from secondary analyses of the same parent cohort. Combined TETON analyses were not treated as independent when individual TETON-1 and TETON-2 data were available.

Data extraction was divided equally between the first and second authors. Each author independently cross-checked the data extracted by the other against the corresponding source publication before the dataset was finalized. Any discrepancies identified during verification were resolved by discussion.

Risk of bias was assessed with RoB 2 for randomized trials. Open-label extension and single-arm studies were assessed with an adapted non-randomized study framework. Post hoc INCREASE analyses were evaluated for shared cohort use, retrospective subgrouping, multiplicity and post-randomization selection.

### 2.4. Statistical Analysis

Random-effects meta-analysis used the DerSimonian–Laird method for tau squared and inverse-variance weighting [32]. Continuous outcomes reported as Hodges–Lehmann location-shift differences were pooled using generic inverse variance, with standard errors derived from reported 95% confidence intervals. Hazard ratios were pooled on the log scale. Safety outcomes were pooled as risk ratios from raw event counts. A continuity correction of 0.5 was planned only if a zero cell was present. Statistical heterogeneity was assessed with Q, tau squared and I squared [33].

The primary pooled analysis included only TETON-1 and TETON-2. INCREASE IPF subgroup FVC data were kept as secondary sensitivity evidence because the population had PH-ILD and follow-up was 16 weeks rather than 52 weeks. Open-label and single-arm datasets were not pooled.

The quantitative synthesis used a two-stage aggregate-data approach: trial-level effect estimates and variances were obtained first, followed by inverse-variance pooling. No standardized mean difference or odds ratio was used. Because only two separately randomized direct-IPF trials within the coordinated TETON program contributed to the pooled analyses, sensitivity analyses compared the primary DerSimonian–Laird random-effects model with fixed-effect inverse-variance and restricted maximum likelihood (REML) random-effects models. Modified Hartung–Knapp inference with REML was examined as a conservative small-k sensitivity analysis. Conventional leave-one-out pooling was not performed because exclusion of either trial leaves only one study; influence was assessed by comparing each trial-specific estimate with the pooled estimate. I squared and tau squared were interpreted cautiously because between-study heterogeneity is imprecisely estimated when k = 2.

### 2.5. Certainty of Evidence

Certainty of evidence for major direct-IPF outcomes was assessed using the GRADE framework across risk of bias, inconsistency, indirectness, imprecision and publication bias [34]. Randomized evidence began at high certainty. The assessment was restricted to TETON-1 and TETON-2; PH-ILD and other supportive evidence was not used to increase certainty for the direct-IPF question. Full domain-level judgments and reasons for downgrading are provided in Supplementary Table S14.

Artificial intelligence (AI) tools were used solely to assist with language editing and improvement of manuscript readability. The authors independently performed, verified, and approved all data extraction, statistical analyses, interpretation of results, and final manuscript content, and take full responsibility for the accuracy and integrity of the work.

## 3. Results

### 3.1. Study Selection

**Figure 1:**
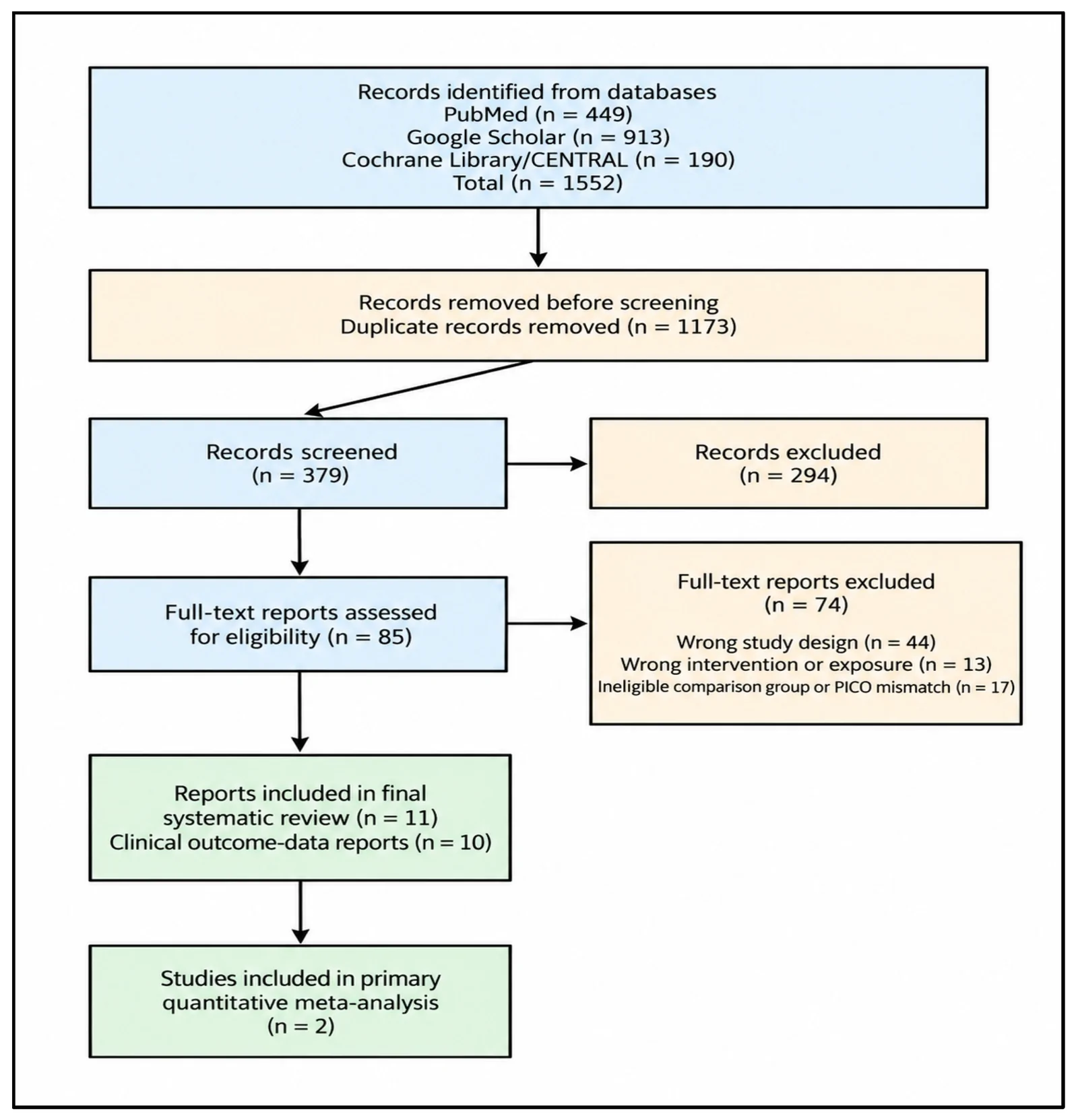
PRISMA flow diagram for study selection.

The database search identified 1552 records. After removal of 1173 duplicate records, 379 records were screened. A total of 294 records were excluded at title and abstract screening. Eighty-five full-text reports were assessed for eligibility. Seventy-four reports were excluded: wrong study design (n = 44), wrong intervention or exposure (n = 13) and ineligible comparison group or PICO mismatch (n = 17). Eleven reports were included in the final systematic review. Ten reports contained clinical outcome data, and one report was retained as a trial design paper, as shown in Figure 1.

### 3.2. Characteristics of Included Studies

The 11 included reports represented four principal patient-level datasets: TETON-1, TETON-2, INCREASE, and the Japanese PH-ILD cohort, as shown in Table 1. TETON-1 and TETON-2 were the two separately randomized direct IPF trials within the coordinated TETON program eligible for the main pooled analysis [29,30]. The INCREASE parent trial and its post hoc analyses shared the same 326-patient dataset and were not counted as independent studies [26,27,35,36,37,38]. The Japanese single-arm study and long-term follow-up represented the same Japanese cohort [39,40].

### 3.3. Baseline and Intervention Characteristics

The direct IPF trials enrolled older and predominantly male populations with preserved to moderately reduced FVC. TETON-1 included 598 patients and TETON-2 included 593 patients. More than three-quarters of patients in both trials were receiving stable background antifibrotic therapy at baseline, as shown in Table 2. Both trials used inhaled treprostinil delivered by the TD-300 ultrasonic nebulizer, starting at 3 breaths four times daily and titrating to 12 breaths four times daily or the highest tolerated dose.

### 3.4. Risk of Bias and Evidence Quality

TETON-1 and TETON-2 were judged as having some concerns under RoB 2 because attrition before week 52 was substantial and sequence generation details were not fully described in the main texts, as shown in Table 3. Central spirometry review, blinded treatment allocation and independent event adjudication strengthened outcome measurement in both trials. INCREASE was also judged as having some concerns because clinical worsening and exacerbation events were investigator reported. The post hoc INCREASE analyses were exploratory because they reused the same parent trial population and often used retrospective subgroup definitions.

### 3.5. Primary Efficacy Outcome: Absolute FVC Change

**Figure 2:**
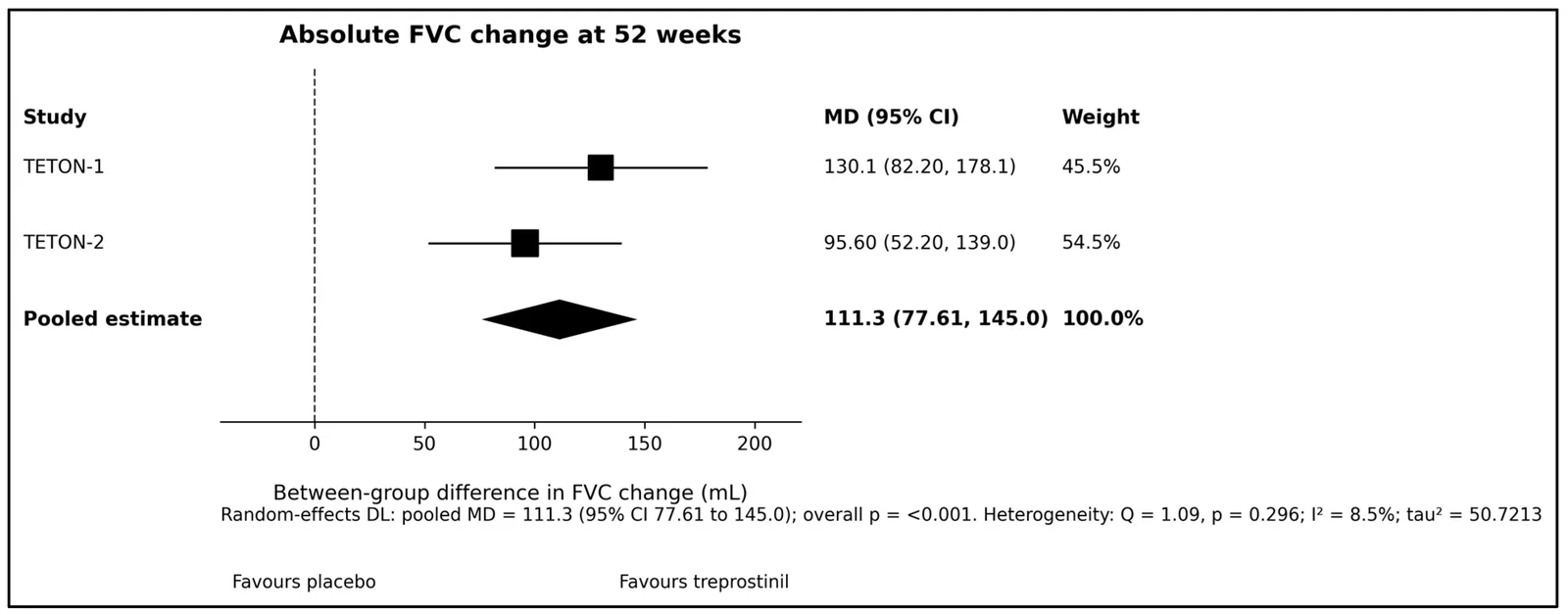
Forest plot for absolute FVC change at 52 weeks.

TETON-1 showed a between-group difference of 130.1 mL in absolute FVC change at week 52, and TETON-2 showed a difference of 95.6 mL, as shown in Figure 2. Random-effects pooling favored inhaled treprostinil with a mean difference of 111.28 mL (95% CI 77.61 to 144.95, *p* < 0.001; I squared 8.5%). The pooled estimate was approximately 111 mL; however, its 95% CI (77.61 to 144.95 mL) spans values below and above the commonly cited approximately 100 mL patient-level threshold, and a between-group treatment effect should not be equated directly with a patient-level minimal clinically important difference [41].

### 3.6. Secondary Lung-Function and Quality-of-Life Outcomes

**Figure 3:**
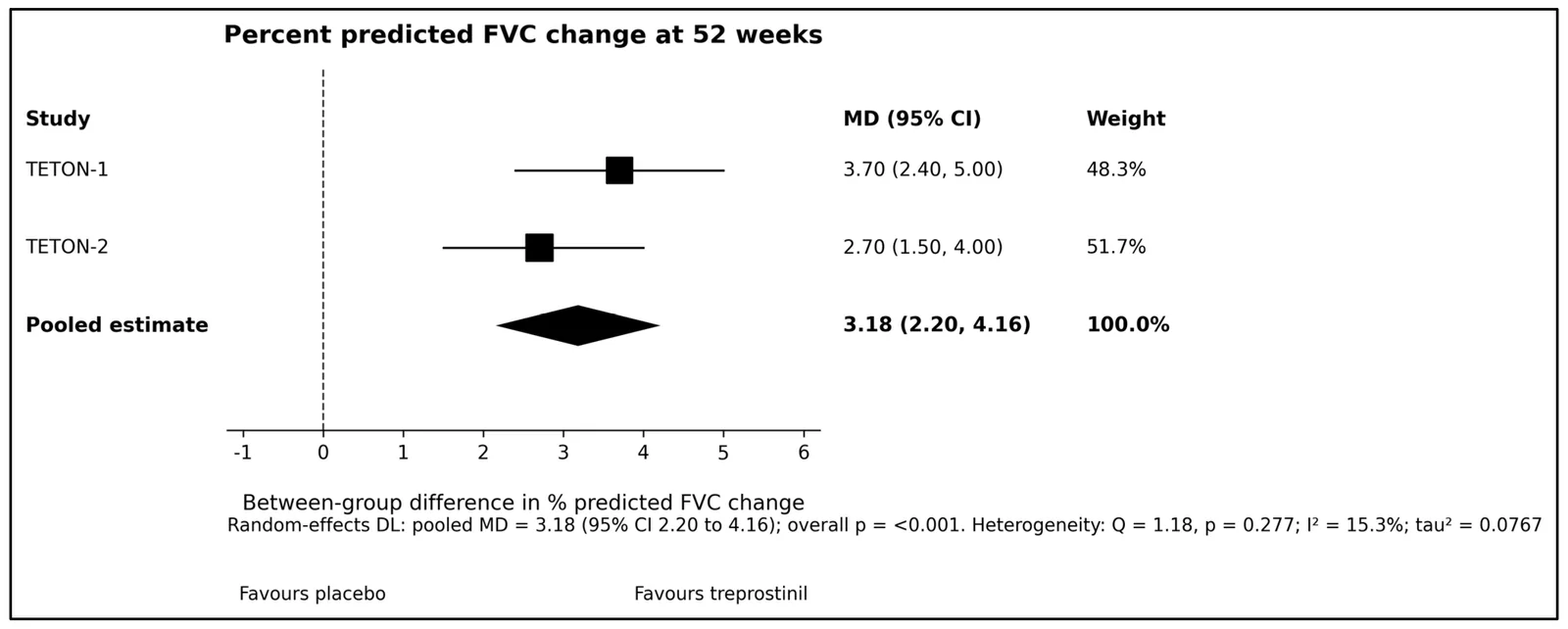
Forest plot for percentage predicted FVC change at 52 weeks.

**Figure 4:**
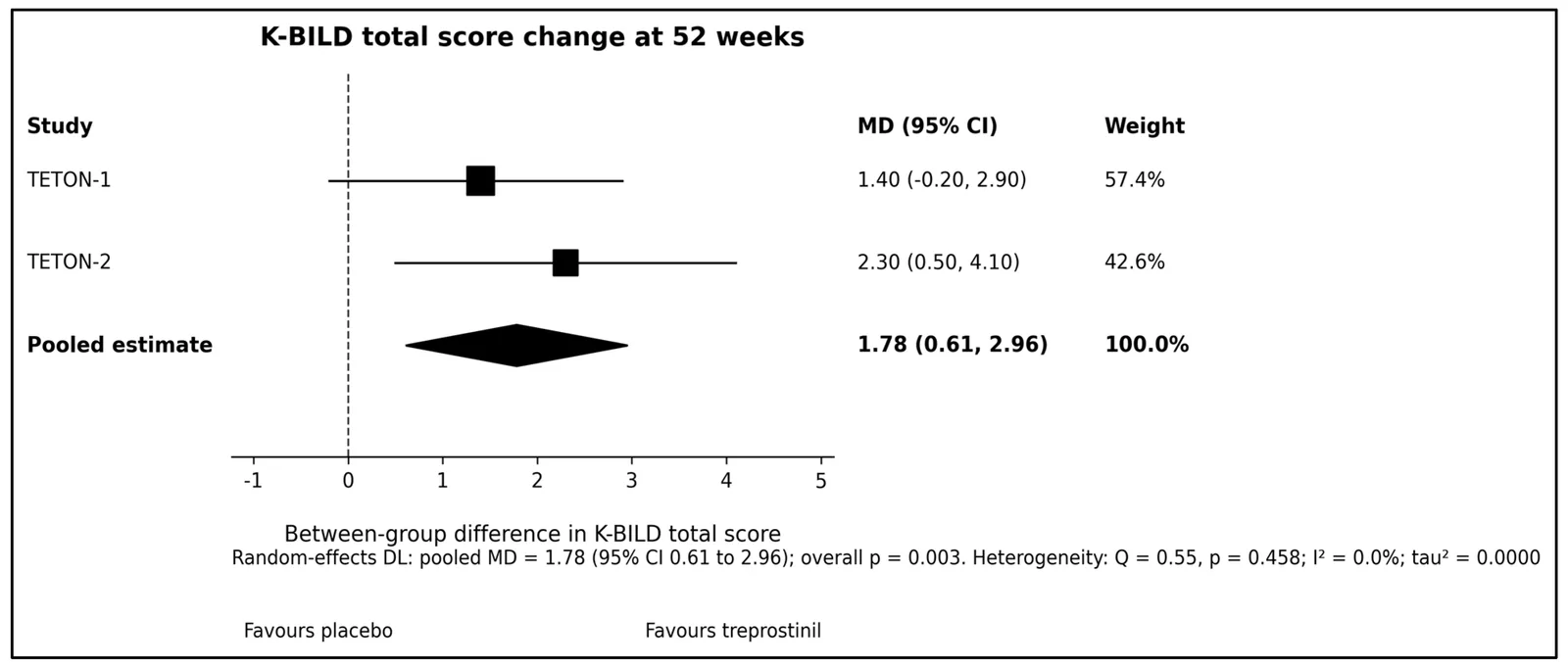
Forest plot for K-BILD total score change at 52 weeks.

**Figure 5:**
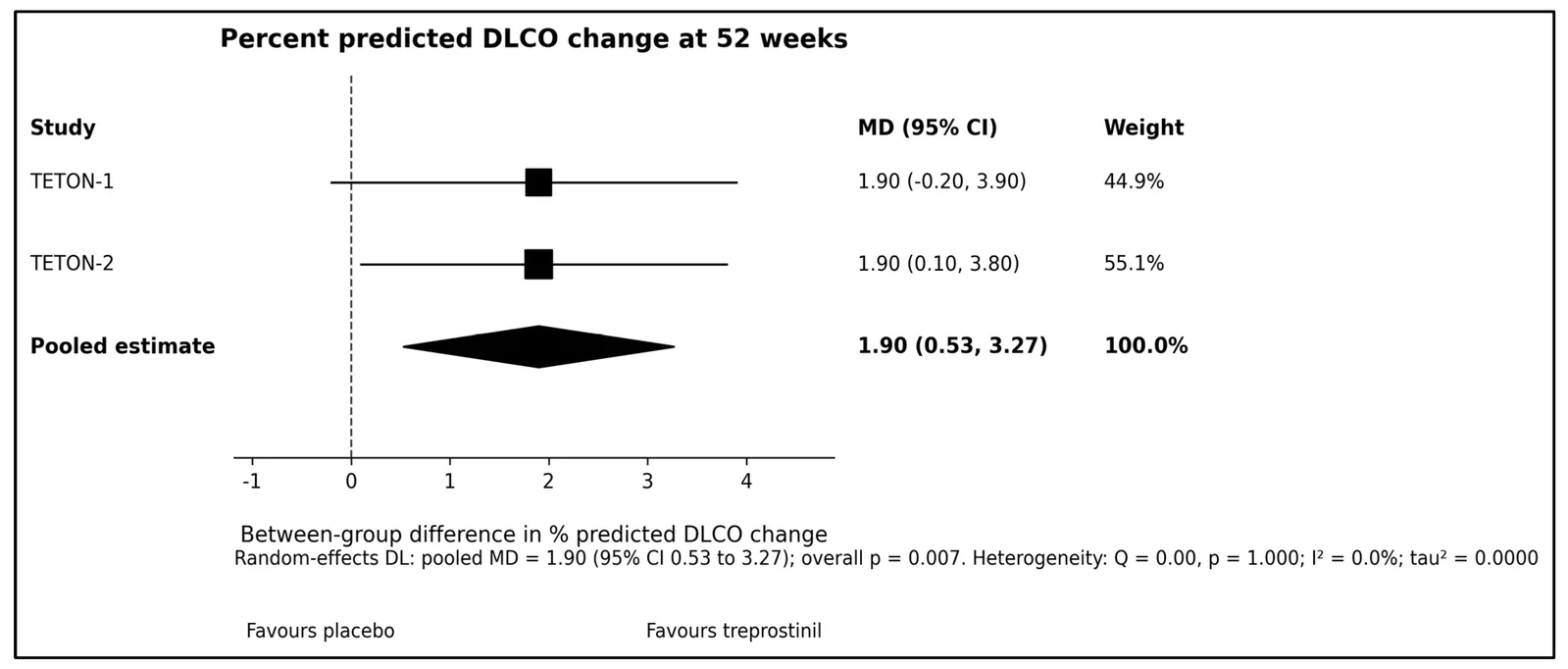
Forest plot for percentage predicted DLCO change at 52 weeks.

Pooled percentage predicted FVC change also favored inhaled treprostinil (MD 3.18%, 95% CI 2.20 to 4.16, *p* < 0.001; I squared 15.3%) Figure 3. K-BILD total score favored treprostinil (MD 1.78, 95% CI 0.61 to 2.96, *p* = 0.003; I squared 0.0%) Figure 4. Percentage predicted DLCO favored treprostinil (MD 1.90%, 95% CI 0.53 to 3.27, *p* = 0.007; I squared 0.0%) Figure 5. These secondary endpoint estimates should be interpreted as nominal pooled findings because the TETON statistical hierarchy stopped after the non-significant acute exacerbation endpoint in each trial. K-BILD interpretation also needs caution because the reported between-group change was below some published thresholds for patient-level meaning [42].

### 3.7. Clinical Worsening, Acute Exacerbation and Mortality

**Figure 6:**
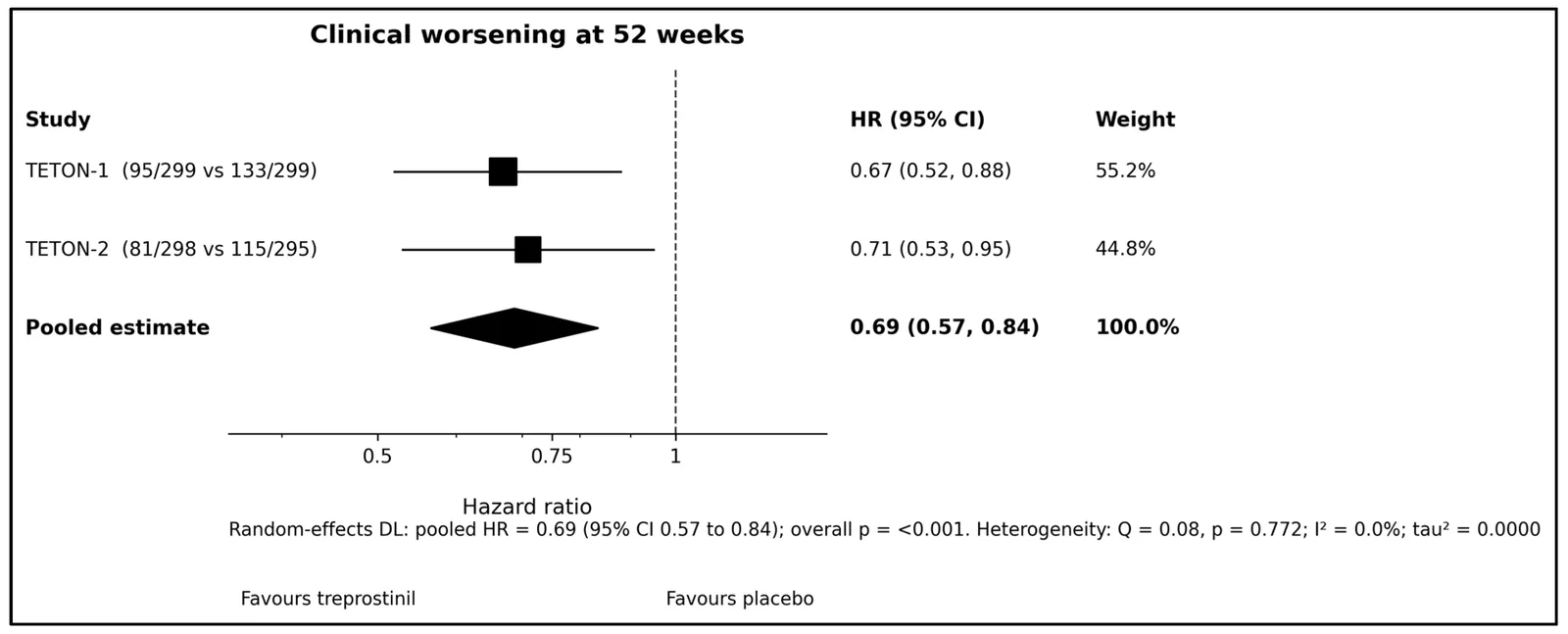
Forest plot for clinical worsening at 52 weeks.

**Figure 7:**
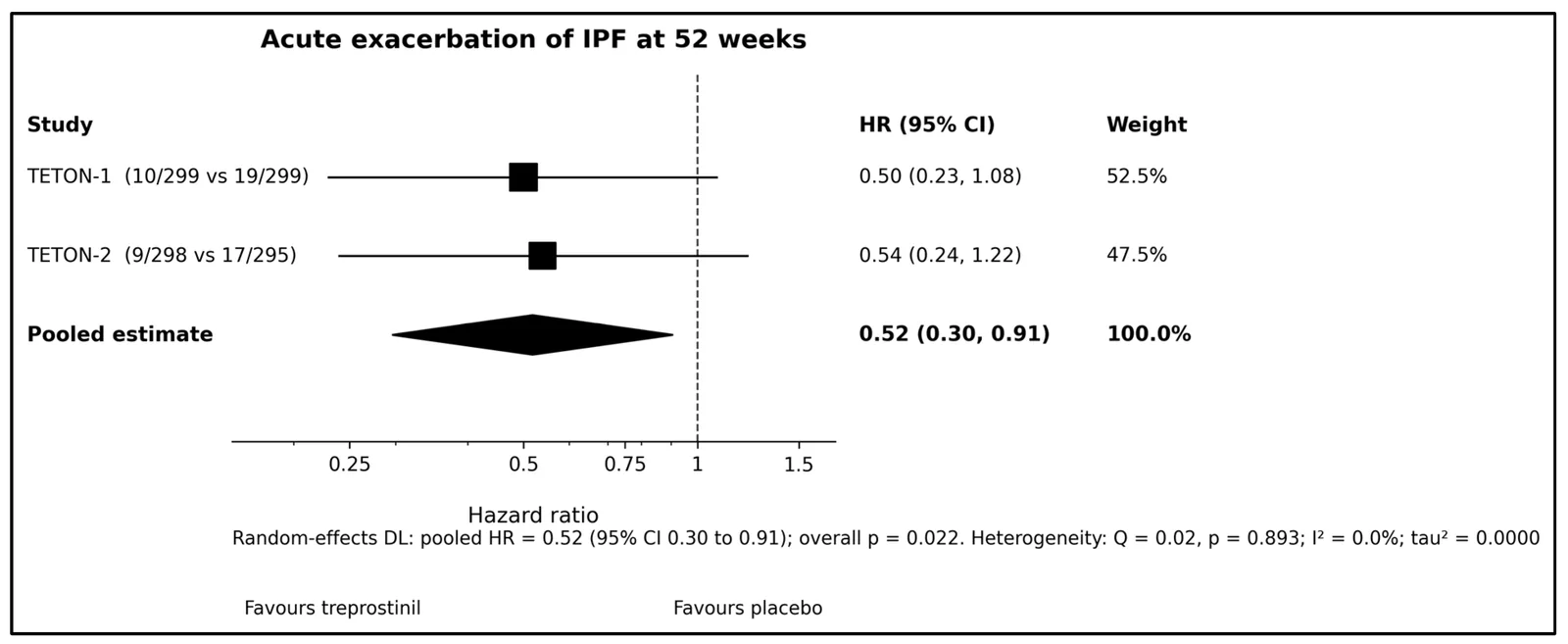
Forest plot for acute exacerbation of IPF at 52 weeks.

**Figure 8:**
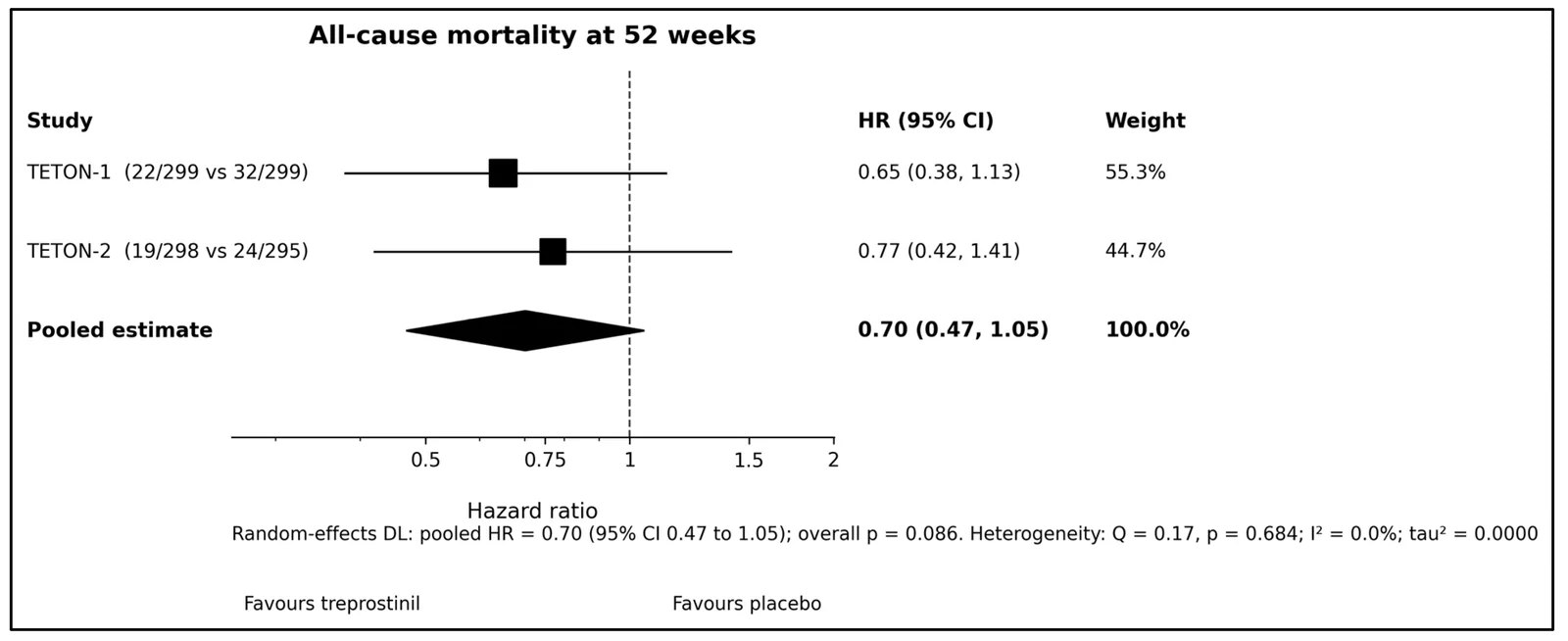
Forest plot for all-cause mortality at 52 weeks.

Clinical worsening was lower with inhaled treprostinil in both TETON trials. Pooled analysis showed in Figure 6 a hazard ratio of 0.69 (95% CI 0.57 to 0.84, *p* < 0.001; I squared 0.0%). Acute exacerbation of IPF was less frequent with treprostinil in pooled analysis (HR 0.52, 95% CI 0.30 to 0.91, *p* = 0.022; I squared 0.0%) Figure 7. All-cause mortality favored treprostinil numerically but did not reach statistical significance (HR 0.70, 95% CI 0.47 to 1.05, *p* = 0.086; I squared 0.0%) Figure 8.

### 3.8. Safety and Tolerability

**Figure 9:**
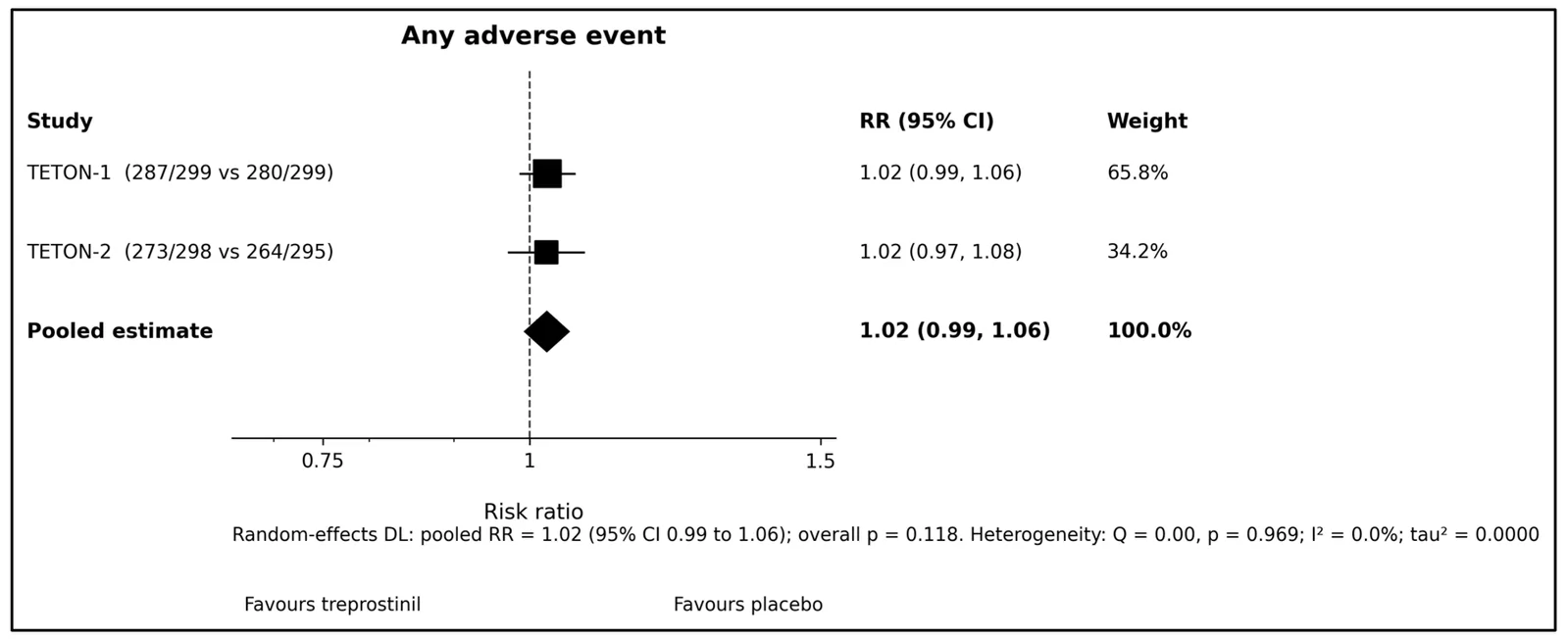
Forest plot for any adverse event.

**Figure 10:**
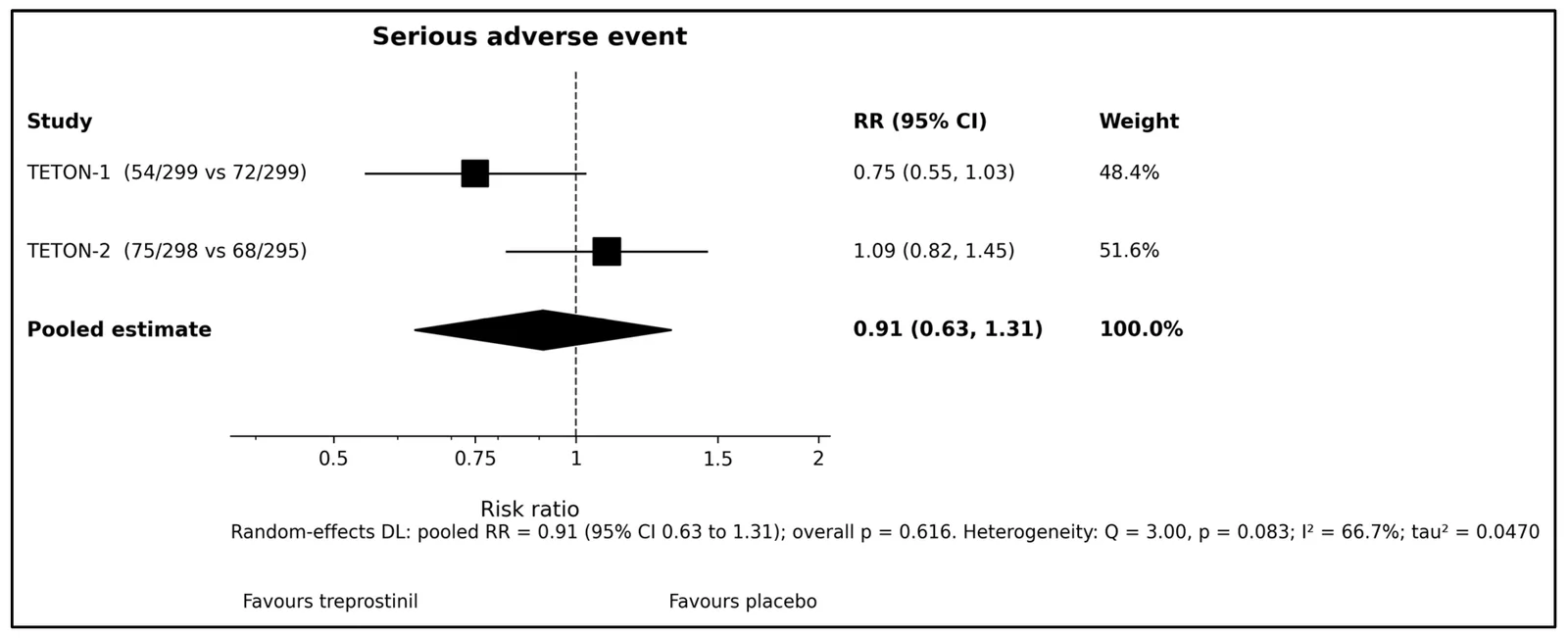
Forest plot for serious adverse event.

**Figure 11:**
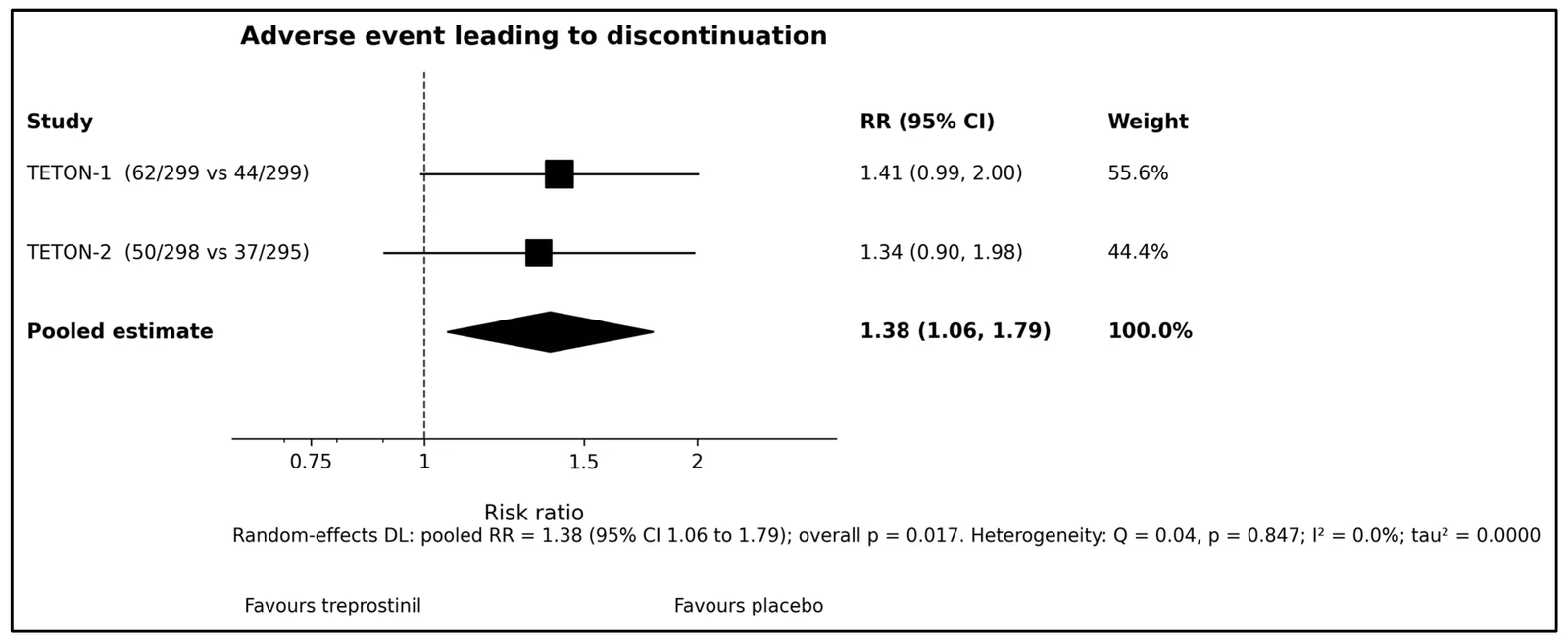
Forest plot for adverse event leading to discontinuation.

Any adverse event was not significantly different between groups (RR 1.02, 95% CI 0.99 to 1.06, *p* = 0.118) Figure 9. Serious adverse events also did not differ significantly (RR 0.91, 95% CI 0.63 to 1.31, *p* = 0.616), Figure 10 but heterogeneity was moderate to high for this endpoint (I squared 66.7%). Adverse events leading to treatment discontinuation were more common with inhaled treprostinil (RR 1.38, 95% CI 1.06 to 1.79, *p* = 0.017) Figure 11, Table 4.

**Figure 12:**
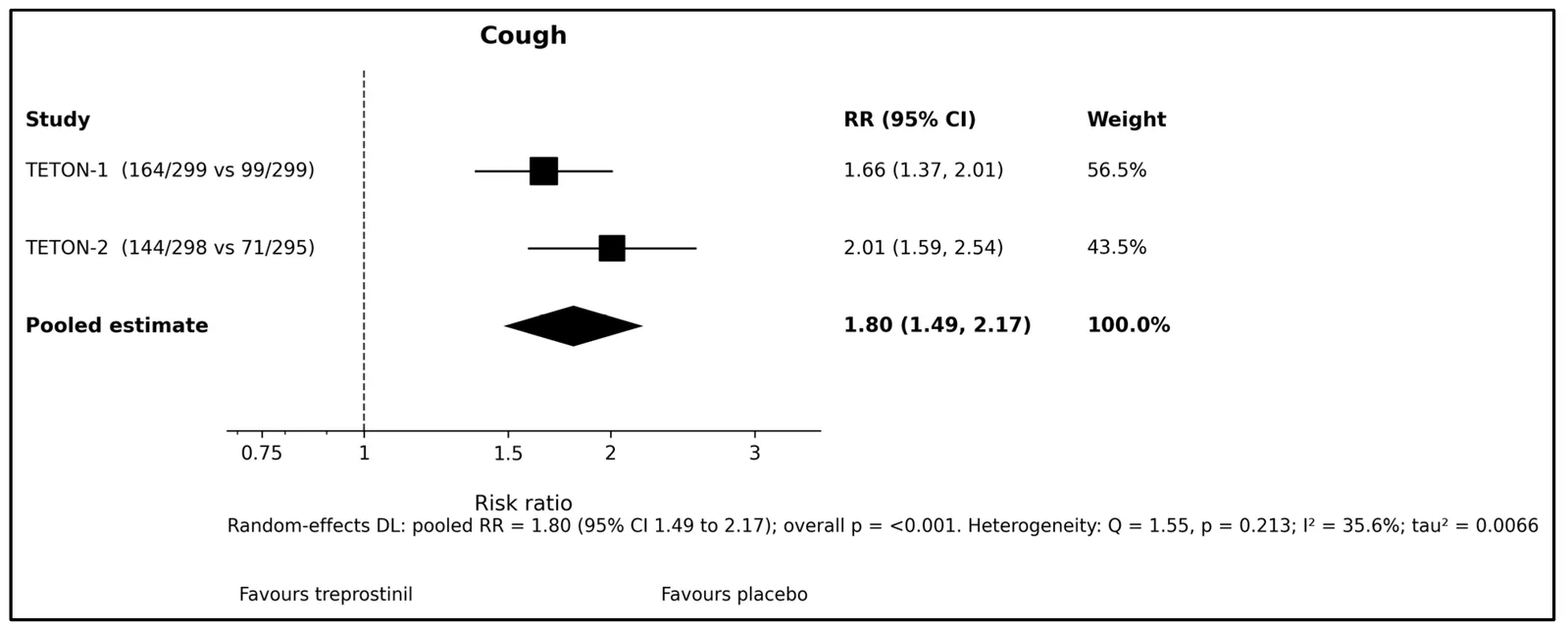
Forest plot for cough.

**Figure 13:**
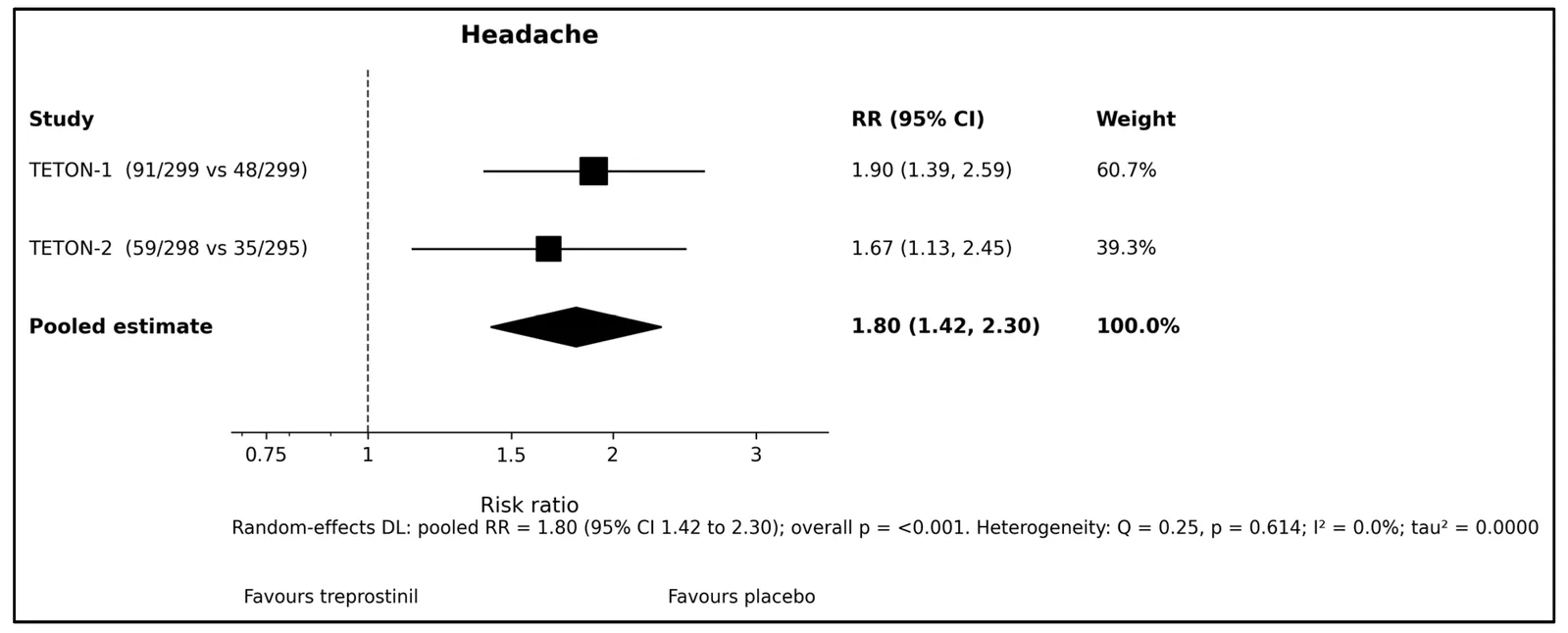
Forest plot for headache.

**Figure 14:**
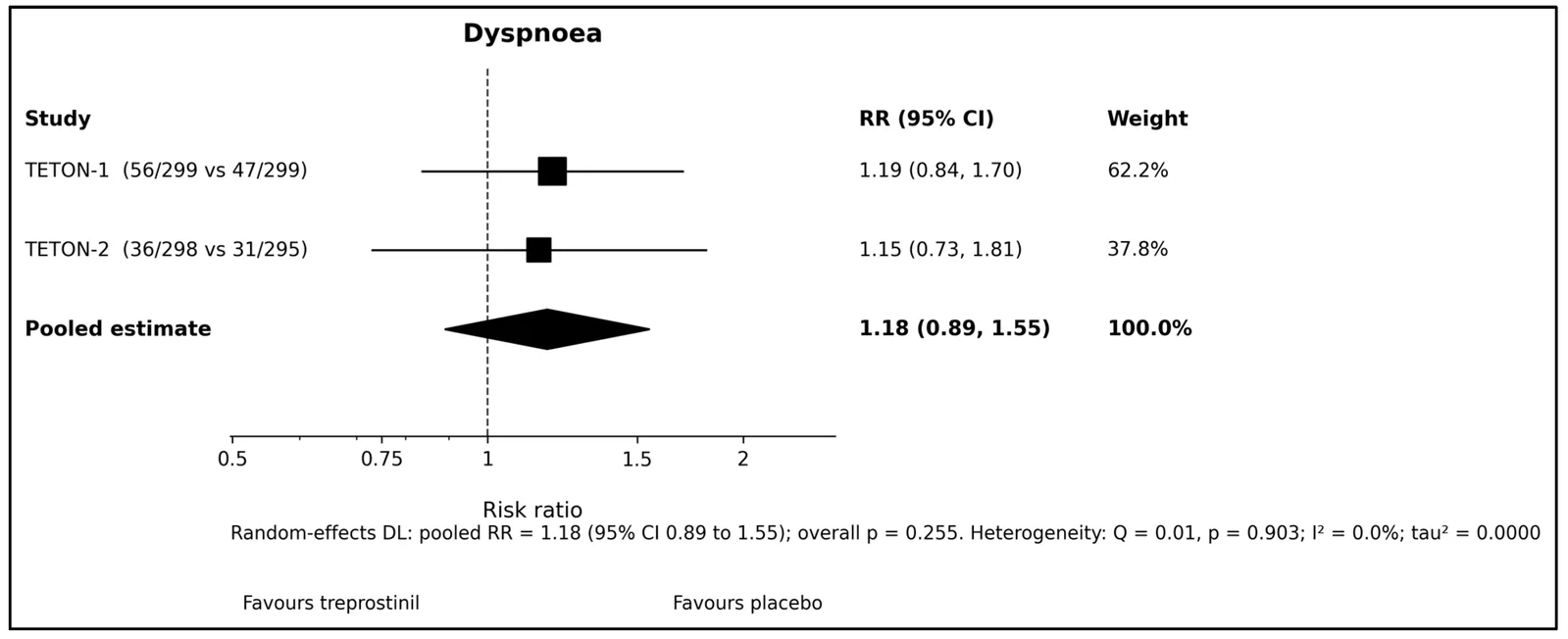
Forest plot for dyspnea.

**Figure 15:**
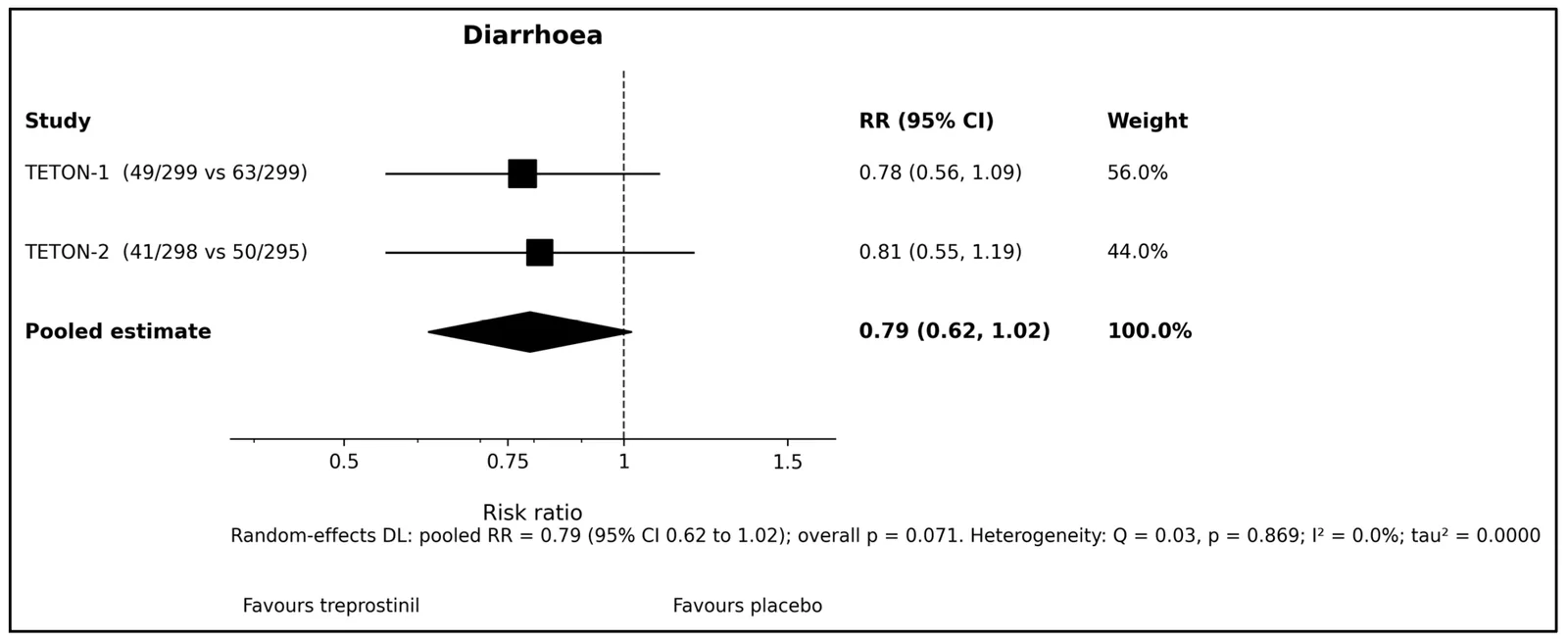
Forest plot for diarrhea.

Cough and headache were the clearest tolerability signals. Cough was more frequent with treprostinil (RR 1.80, 95% CI 1.49 to 2.17, *p* < 0.001; I squared 35.6%) Figure 12. Headache was also more common (RR 1.80, 95% CI 1.42 to 2.30, *p* < 0.001; I squared 0.0%) Figure 13. Dyspnea and diarrhea were not significantly different between groups Figure 14 and Figure 15.

### 3.9. Secondary and Narrative Evidence

The INCREASE IPF subgroup supported an FVC signal at 16 weeks, with a placebo-corrected absolute FVC difference of 168.5 mL (95% CI 40.1 to 297.0, *p* = 0.011) and a percentage predicted FVC difference of 3.5% (95% CI 0.7 to 6.3, *p* = 0.015) [27]. These data were not pooled with TETON-1 and TETON-2 because INCREASE enrolled patients with PH-ILD, not direct IPF, and used 16-week follow-up. Six-minute walk distance was treated as supportive exercise-capacity evidence because its measurement properties have been described in IPF Table 5 [43].

The parent INCREASE trial showed improvement in peak 6 min walk distance by 31.12 m at 16 weeks, reduced NT-proBNP and prolonged time to clinical worsening in PH-ILD [26]. Post hoc INCREASE analyses suggested fewer disease-progression events, better outcomes in patients who reached higher inhaled doses and benefit across less severe hemodynamic strata [35,36,38]. These findings were hypothesis-generating because they were derived from the same parent dataset.

The INCREASE open-label extension and Japanese PH-ILD studies provided long-term and non-randomized support for feasibility and physiological activity [37,39,40]. The Japanese single-arm study reported PVRI reduction of −40.1% at week 16. The long-term Japanese follow-up described preservation of functional class in many patients and death- or transplant-free survival of 100.0% at 1 year, 93.8% at 2 years and 75.0% at 3 years. These data were not pooled because they lacked a concurrent control group.

Table 6 depicts the summary of pooled results from all the studies. 

### 3.10. Sensitivity Analyses and Certainty of Evidence

Across all 14 pooled outcomes, fixed-effect and REML sensitivity analyses produced point estimates that were essentially unchanged from the primary DerSimonian–Laird analysis. For absolute FVC change, the pooled estimates were MD 111.28 mL (95% CI 77.61 to 144.95) with DerSimonian–Laird, MD 111.14 mL (95% CI 78.96 to 143.31) with fixed-effect inverse variance and MD 111.28 mL (95% CI 77.61 to 144.95) with REML. Modified Hartung–Knapp inference produced substantially wider intervals with only two studies; the corresponding FVC estimate was MD 111.28 mL (95% CI −106.99 to 329.56). For clinical worsening, the point estimate remained HR 0.69 across conventional models, while the modified Hartung–Knapp 95% CI was 0.19 to 2.44. The trial-specific estimates for both FVC and clinical worsening were directionally concordant. These analyses indicate stability of the pooled point estimates across conventional models but substantial uncertainty in small-k inference. Full sensitivity results are provided in Supplementary Table S13.

GRADE certainty was moderate for absolute FVC change and clinical worsening, low for acute exacerbation and all-cause mortality, low for serious adverse events and moderate for adverse events leading to treatment discontinuation Table 7. The principal reasons for downgrading were imprecision with only two trials, sparse events for acute exacerbation and mortality, and inconsistency for serious adverse events.

## 4. Discussion

This meta-analysis found that inhaled treprostinil preserved FVC in IPF across two separately randomized phase 3 trials within the coordinated TETON program. The pooled 52-week effect of 111.28 mL was consistent across TETON-1 and TETON-2, with low heterogeneity. The direction and size of effect were also concordant for percentage predicted FVC and DLCO. The result is clinically relevant because FVC decline is linked to mortality and has long been used as a principal endpoint in IPF trials [6,7,8,41]. The conventional pooled estimate was stable across fixed-effect and REML analyses, but the modified Hartung–Knapp interval was much wider with only two studies. The 111 mL between-group effect therefore supports preservation of FVC at the trial level but should not be interpreted as proof that every treated patient achieves a clinically meaningful improvement.

The clinical worsening result provides a second line of trial-level support. Both TETON trials used a composite that included death, respiratory hospitalization and relative decline of at least 10% predicted FVC. The pooled hazard ratio was 0.69. This effect aligns with the FVC findings and makes a pure spirometric artifact less likely. Acute exacerbation events favored treprostinil in the pooled analysis. The individual trials still did not reach formal significance for this hierarchical endpoint. Mortality moved in the same direction but the estimate was not statistically significant. The most defensible reading is narrower: treprostinil reduces lung-function decline and clinical worsening. A mortality benefit is not proven at 52 weeks.

The safety signal is less simple. The incidence of serious adverse events were not increased in the pooled analysis. Any adverse event were also similar between the arms. Discontinuation due to adverse events was higher with treprostinil. Cough and headache were more frequent. This pattern is plausible for an inhaled prostacyclin. It is also clinically important. A treatment that preserves physiology in a trial still depends on whether patients can continue it outside trial conditions.

The INCREASE study helps to explain the biological basis for TETON but it should not be included into the same evidence stream. INCREASE enrolled PH-ILD patients and measured 6 min walk distance over 16 weeks [26]. The IPF subgroup FVC finding was striking. It was still a post hoc subgroup result based on observed data at week 16 [27]. TETON-1 and TETON-2 asked the more direct question: does inhaled treprostinil preserve FVC in IPF over 52 weeks [29,30]? The randomized direct IPF evidence answers is yes.

These results also affect how pulmonary vascular pathways are viewed in fibrotic lung disease. Earlier vasodilator trials in idiopathic interstitial pneumonia-associated pulmonary hypertension and fibrotic idiopathic interstitial pneumonia did not support broad use [19,20]. Inhaled treprostinil is different in route, pharmacology and trial signal. The TETON findings point to possible prostacyclin pathway effects beyond pulmonary vasodilation. Experimental work on fibroblast biology and extracellular matrix pathways supports that interpretation [22,23,24,25].

Treprostinil should be considered complimentary to antifibrotic therapy in the current armamentarium, not replace it. Pirfenidone and nintedanib reduce FVC decline in IPF [10,11,12]. Trials in progressive fibrosing ILD and systemic sclerosis-associated ILD show that antifibrotic treatment can work across fibrotic phenotypes [44,45,46]. Most TETON participants were already on background antifibrotics. Treprostinil therefore is more appropriate as an additive to contemporary care rather than comparative against untreated IPF. Sequencing and combination strategy still need direct evidence, as well as long-term persistence. More recently, FIBRONEER-IPF showed that nerandomilast reduced 52-week FVC decline versus placebo, further expanding the therapeutic landscape in IPF [47].

This review has one main strength: it keeps the evidence streams separate. The two separately randomized direct-IPF TETON trials were analyzed as the primary evidence. Combined TETON analyses were not counted again. INCREASE post hoc publications were not double-counted. Safety outcomes came from the same TETON trials that drove the efficacy result. This approach limits inflated precision. The TETON program had already reported a prespecified combined analysis [29]. The added value of this review is therefore the formal outcome-specific synthesis across the broader evidence base, including safety pooling, small-k sensitivity analyses, GRADE certainty assessment and explicit separation of direct-IPF evidence from overlapping or indirect PH-ILD evidence.

The limitations are important to consider during interpretations of this review’s findings. Only two direct IPF trials were available for meta-analysis. Heterogeneity estimates are therefore unstable. TETON continuous outcomes were reported as Hodges–Lehmann location-shift estimates rather than conventional mean differences. Generic inverse-variance pooling fits the reported data but it cannot replace patient-level analysis. Attrition was substantial in the TETON trials. Secondary endpoint inference after the failed acute exacerbation hierarchy should be treated as nominal. Embase was not searched because access was unavailable. PubMed, Google Scholar and Cochrane/CENTRAL searches were supplemented by focused verification searches. Google Scholar has limited reproducibility because its result ranking is dynamic, and the absence of Embase further limits search coverage. The protocol itself was finalized before screening, but PROSPERO registration was retrospective rather than prospective. Fixed-effect and REML analyses yielded similar point estimates, whereas modified Hartung–Knapp inference produced markedly wider confidence intervals, emphasizing the uncertainty inherent in random-effects inference based on only two studies.

Future studies should identify which IPF patients benefit most from inhaled treprostinil. They should test whether benefit differs by background antifibrotic therapy. Earlier initiation may matter for exacerbation or survival and needs direct assessment. Cough-related discontinuation also needs a practical mitigation strategy. Patient-level meta-analysis would allow for a better assessment of disease severity, antifibrotic background therapy, achieved dose and regional variation.

## 5. Conclusions

Inhaled treprostinil preserved FVC and reduced clinical worsening in adults with IPF across two separately randomized phase 3 trials within the coordinated TETON program. The pooled safety data did not show a significant increase in serious adverse events, but treatment discontinuation, cough and headache were more frequent. INCREASE IPF-PH subgroup data, open-label extension data and Japanese PH-ILD studies support the biological and clinical rationale, but they should stay separate from the primary direct-IPF meta-analysis. These conclusions remain limited by the availability of only two direct-IPF randomized trials and do not establish a mortality benefit or a disease-modifying antifibrotic effect.

## Figures and Tables

**Table 1:** Characteristics and analytical role of included reports.

Study	Region	Design	Population	N	Arms	Follow-Up	Role
TETON-1	United States and Canada	Phase 3 double-blind RCT	Direct IPF	598	299/299	52 weeks	Main meta-analysis
TETON-2	Asia-Pacific, Europe and South America	Phase 3 double-blind RCT	Direct IPF	593	298/295	52 weeks	Main meta-analysis
INCREASE	United States	Phase 3 double-blind RCT	PH-ILD; IPF subgroup n = 92	326	163/163	16 weeks	Secondary and sensitivity
INCREASE FVC post hoc	United States	Post hoc RCT analysis	PH-ILD; IPF subgroup n = 92	326	163/163	16 weeks	Secondary and narrative
INCREASE disease progression	United States	Post hoc RCT analysis	PH-ILD	326	163/163	16 weeks	Narrative only
INCREASE dosage	United States	Post hoc RCT analysis	PH-ILD	302	149/153	16 weeks	Narrative only
INCREASE OLE	United States	Open-label extension	PH-ILD	242	242/NA	Median exposure 62.1 weeks	Narrative only
INCREASE less severe hemodynamics	United States and Canada	Post hoc RCT analysis	PH-ILD	326	163/163	16 weeks; OLE to 1 year	Narrative only
Japanese PH-ILD study	Japan	Prospective single-arm study	PH-ILD; IPF subgroup n = 7	20	20/NA	52 weeks	Narrative only
Japanese long-term follow-up	Japan	Open-label long-term follow-up	PH-ILD	20	20/NA	Median 107.5 weeks	Narrative only
TETON design paper	International	Trial protocol/design	Direct IPF trial design	NA	NA	NA	Design background only

IPF—Idiopathic pulmonary fibrosis; RCT—Randomized controlled trial; PH-ILD—Pulmonary hypertension associated with Interstitial lung disease.

**Table 2:** Baseline and intervention characteristics of principal datasets.

Dataset	Mean Age	Male	Baseline FVC	Baseline DLCO	Background Antifibrotic Use	Intervention
TETON-1	73.0	77.3%	74.6%	NR	77.6%	Three breaths [18 micrograms] four times daily, titrated to 12 breaths [72 micrograms] four times daily
TETON-2	71.7	80.1%	76.8%	NR	75.4%	Same as TETON-1
INCREASE	66.5	54.0%	62.5%	NR	Variable background therapy	Three breaths four times daily, titrated to 9 to 12 breaths four times daily
Japanese PH-ILD study	NR	NR	NR	NR	NR	Inhaled treprostinil, titrated to tolerated dose

ILD—Interstitial lung Disease; FVC—Forced vital capacity; DLCO—Diffusing capacity of lungs for carbon monoxide.

**Table 3:** Risk of bias and methodological quality summary.

Dataset or Analysis	Tool	Judgment	Main Reason
TETON-1	RoB 2	Some concerns	High attrition before week 52 and incomplete sequence details in the main text; central spirometry and blinded event adjudication supported outcome measurement.
TETON-2	RoB 2	Some concerns	High attrition before week 52 and incomplete sequence details in the main text; sensitivity analyses supported the primary result.
INCREASE	RoB 2	Some concerns	Central randomization and double-blinding were used; clinical worsening and exacerbations were investigator-reported without independent adjudication.
INCREASE post hoc analyses	Methodological critique	Exploratory	Shared parent dataset, retrospective subgrouping, no multiplicity control and some post-randomization grouping.
INCREASE OLE	Adapted NOS	Low to moderate	Single-arm open-label design, no concurrent comparator and high discontinuation.
Japanese PH-ILD studies	Adapted NOS	Low to moderate	Small single-arm cohort, no comparator and survivor effects during long-term follow-up.

OLE—Open label extension; PH-ILD—Pulmonary hypertension associated with Interstitial lung disease.

**Table 4:** Safety event counts from the direct IPF randomized trials.

Study	Safety Outcome	Treprostinil	Placebo
TETON-1	Any AE	287/299	280/299
TETON-1	Serious AE	54/299	72/299
TETON-1	AE leading to discontinuation	62/299	44/299
TETON-1	Death	22/299	32/299
TETON-1	Cough	164/299	99/299
TETON-1	Headache	91/299	48/299
TETON-1	Dyspnea	56/299	47/299
TETON-1	Diarrhea	49/299	63/299
TETON-2	Any AE	273/298	264/295
TETON-2	Serious AE	75/298	68/295
TETON-2	AE leading to discontinuation	50/298	37/295
TETON-2	Death	19/298	24/295
TETON-2	Cough	144/298	71/295
TETON-2	Headache	59/298	35/295
TETON-2	Dyspnea	36/298	31/295
TETON-2	Diarrhea	41/298	50/295
TETON-2	Dizziness	29/298	19/295
TETON-2	Nausea	24/298	26/295
TETON-2	Throat irritation or pharyngo-laryngeal pain	21/298	7/295

**Table 5:** Secondary, sensitivity and narrative evidence not pooled in the primary analysis.

Dataset	Outcome	Effect	95% CI	*p*-Value	Role
INCREASE IPF subgroup	Absolute FVC at week 16	168.5 mL	40.1 to 297.0	0.011	Secondary sensitivity only
INCREASE IPF subgroup	% predicted FVC at week 16	3.5%	0.7 to 6.3	0.015	Secondary sensitivity only
INCREASE overall PH-ILD	6MWD at week 16	31.12 m	16.85 to 45.39	<0.001	PH-ILD evidence only
INCREASE disease progression	Time to first progression event	HR 0.71	0.54 to 0.94	0.019	Narrative only
INCREASE dosage	Clinical worsening from weeks 4 to 16	17.1% in high-dose treprostinil vs. about 34% in placebo groups	NR	0.006	Narrative only
INCREASE less severe hemodynamics	Exacerbations in PVR <= 5 WU subgroup	HR 0.43	0.23 to 0.81	0.01	Narrative only
INCREASE OLE	6MWD change from RCT baseline to OLE week 52	3.5 m	NR	NR	Narrative only
Japanese PH-ILD study	PVRI change at week 16	−40.1%	−53.1 to −27.2	NR	Narrative only
Japanese long-term follow-up	Death- or transplant-free survival	100.0% at 1 year, 93.8% at 2 years and 75.0% at 3 years	NR	NR	Narrative only

6MWD—Six-minute walk distance; OLE- Open label extension; PH-ILD—Pulmonary hypertension associated with Interstitial lung disease; RCT—Randomized controlled trial; PVR—Pulmonary vascular resistance.

**Table 6:** Pooled random-effects meta-analysis results.

Fig	Outcome	Measure	Pooled Effect	95% CI	*p*-Value	I Squared	Tau Squared	Interpretation
2	Absolute FVC change	MD	111.28 mL	77.61 to 144.95	<0.001	8.5%	50.7213	Favors treprostinil
3	% predicted FVC change	MD	3.18%	2.20 to 4.16	<0.001	15.3%	0.0767	Favors treprostinil
4	K-BILD total score	MD	1.78	0.61 to 2.96	0.003	0.0%	0.00	Favors treprostinil
5	% predicted DLCO change	MD	1.90%	0.53 to 3.27	0.007	0.0%	0.00	Favors treprostinil
6	Clinical worsening	HR	0.69	0.57 to 0.84	<0.001	0.0%	0.00	Favors treprostinil
7	Acute exacerbation of IPF	HR	0.52	0.30 to 0.91	0.022	0.0%	0.00	Favors treprostinil
8	All-cause mortality	HR	0.70	0.47 to 1.05	0.086	0.0%	0.00	Not significant
9	Any adverse event	RR	1.02	0.99 to 1.06	0.118	0.0%	0.00	Not significant
10	Serious adverse event	RR	0.91	0.63 to 1.31	0.616	66.7%	0.0470	Not significant
11	AE leading to discontinuation	RR	1.38	1.06 to 1.79	0.017	0.0%	0.00	Higher with treprostinil
12	Cough	RR	1.80	1.49 to 2.17	<0.001	35.6%	0.0066	Higher with treprostinil
13	Headache	RR	1.80	1.42 to 2.30	<0.001	0.0%	0.00	Higher with treprostinil
14	Dyspnea	RR	1.18	0.89 to 1.55	0.255	0.0%	0.00	Not significant
15	Diarrhea	RR	0.79	0.62 to 1.02	0.071	0.0%	0.00	Not significant

**Table 7:** GRADE certainty of evidence for major direct-IPF outcomes.

Outcome	Pooled Effect	Main GRADE Limitation	Certainty	Interpretation
Absolute FVC change at 52 weeks	MD 111.28 mL (95% CI 77.61 to 144.95)	Imprecision: CI spans values below and above a commonly cited ~100 mL clinical threshold; small-k adjusted inference was wide.	Moderate	Greater preservation of FVC, with uncertainty about the magnitude of clinical importance.
Clinical worsening at 52 weeks	HR 0.69 (95% CI 0.57 to 0.84)	Imprecision: only two trials; modified Hartung–Knapp interval crossed the null.	Moderate	Lower hazard of clinical worsening, but certainty is limited by small-k inference.
Acute exacerbation of IPF	HR 0.52 (95% CI 0.30 to 0.91)	Very serious imprecision: sparse events and very wide small-k adjusted interval.	Low	Possible reduction; magnitude remains uncertain.
All-cause mortality	HR 0.70 (95% CI 0.47 to 1.05)	Very serious imprecision: CI includes no effect and clinically important benefit; events were sparse.	Low	Mortality benefit is not established.
Serious adverse events	RR 0.91 (95% CI 0.63 to 1.31)	Inconsistency (I squared 66.7%) and imprecision.	Low	No clear difference; important benefit or harm cannot be excluded.
AE leading to discontinuation	RR 1.38 (95% CI 1.06 to 1.79)	Imprecision: lower CI bound is close to the null and small-k adjusted inference was wide.	Moderate	Treatment discontinuation due to adverse events is probably more frequent with treprostinil.

## Data Availability

The data supporting the findings of this study are available from the corresponding author upon reasonable request. This study is a systematic review and meta-analysis based exclusively on data from previously published studies. No individual participant-level data were collected or generated. All data analyzed are available in the published articles and Supplementary Materials cited in this manuscript.

## Supplementary Materials

The following supporting information can be downloaded at https://www.mdpi.com/article/10.3390/arm94050073/s1: Supplementary Figure S1: Forest plots of the pooled efficacy and safety outcomes; Supplementary Tables S1–S14: search strategies, study selection, study characteristics, data extraction, risk of bias, pooled analyses, sensitivity analyses, effect measure definitions, outcome-specific interpretations, and GRADE certainty-of-evidence assessment. References [1,2,3,4,5,6,7,8,9,10,11,12,13,14,15,16,17,18,19,20,21,22,23,24,25,26,27,28,29,30,31,32,33,34,35,36,37,38,39,40,41,42,43,44,45,46,47] are cited in the Supplementary Materials.
