# Supplementary material for: Efficacy and Safety of Inhaled Treprostinil in Idiopathic Pulmonary Fibrosis: A Systematic Review and Meta-Analysis
Source: Adv Respir Med. 2026 Oct 8;94(5):73. doi: 10.3390/arm94050073 (PMC13701988; doi:10.3390/arm94050073)
Supplement: Supplementary file 1 [file arm-94-00073-s001.zip › arm-4541865-supplementary.pdf]

Supplementary Data
Efficacy and Safety of Inhaled Treprostinil in Idiopathic Pulmonary Fibrosis

This supplementary document provides the audit trail for database searching, study classification, extraction decisions, risk-of-bias assessment, meta-analysis-ready data, and citation validation rules [147]. It also documents the exact search strategies and final search date, protocol chronology and reviewer workflow, model-based sensitivity analyses, and GRADE certainty-of-evidence judgments added during peer-review revision.

Supplementary Methods S1. Search strategy and record counts

Supplementary Table S1. Search strategy and record counts.

Database	Search label	Strategy	Records
PubMed	Main search	Exact PubMed main strategy reproduced in Supplementary Search Appendix S1A; final search run 3 July 2026.	449
PubMed	Focused IPF	Exact PubMed focused IPF strategy reproduced in Supplementary Search Appendix S1A; verification search.	33
PubMed	Focused PH-ILD	Exact PubMed focused PH-ILD strategy reproduced in Supplementary Search Appendix S1A; verification search.	87
Google Scholar	Main search	"inhaled treprostinil" "idiopathic pulmonary fibrosis"; Google Scholar custom range 2000-2026.	913
Google Scholar	PH-ILD search	"inhaled treprostinil" "pulmonary hypertension" "interstitial lung disease"; custom range 2000-2026; verification search.	1370
Google Scholar	TETON search	treprostinil TETON idiopathic pulmonary fibrosis; custom range 2000-2026; verification search.	161
Google Scholar	INCREASE FVC search	treprostinil INCREASE forced vital capacity interstitial lung disease pulmonary hypertension; custom range 2000-2026; verification search.	1300
Cochrane Library/CENTRAL	Main broad search	Exact Cochrane main broad strategy reproduced in Supplementary Search Appendix S1A.	190
Cochrane Library/CENTRAL	Focused IPF search	Exact Cochrane focused IPF strategy reproduced in Supplementary Search Appendix S1A; verification search.	18 trials

Database	Search label	Strategy	Records
Cochrane Library/CENTRAL	Focused PH-ILD search	Exact Cochrane focused PH-ILD strategy reproduced in Supplementary Search Appendix S1A; verification search.	57 trials

Supplementary Search S1A. Exact database search strategies

Final search run date: 3 July 2026. The focused searches below were verification searches and were not added to the PRISMA identification total. TETON-2 and TETON-1 had been published online ahead of issue on 11 March 2026 and 18 May 2026, respectively, and were available before the final search; both later appeared in the 9 July 2026 issue. The PubMed strings contained an upper publication-date syntax of 31 December 2026, but because the searches were executed on 3 July 2026, no records indexed after the run date could have been retrieved.

PubMed main search (449 records)

(("treprostinil"[MeSH Terms] OR treprostinil[tiab] OR "inhaled treprostinil"[tiab] OR Tyvaso[tiab] OR "Tyvaso DPI"[tiab]) AND ("idiopathic pulmonary fibrosis"[MeSH Terms] OR "idiopathic pulmonary fibrosis"[tiab] OR IPF[tiab] OR "interstitial lung disease"[MeSH Terms] OR "interstitial lung disease"[tiab] OR "interstitial lung diseases"[tiab] OR ILD[tiab] OR "pulmonary fibrosis"[tiab] OR "progressive pulmonary fibrosis"[tiab] OR "pulmonary hypertension"[MeSH Terms] OR "pulmonary hypertension"[tiab] OR "PH-ILD"[tiab] OR "pulmonary hypertension associated with interstitial lung disease"[tiab] OR "group 3 pulmonary hypertension"[tiab])) AND ("2000/01/01"[Date - Publication] : "2026/12/31"[Date - Publication])

PubMed focused IPF search (33 records)

(("treprostinil"[MeSH Terms] OR treprostinil[tiab] OR "inhaled treprostinil"[tiab] OR Tyvaso[tiab]) AND ("idiopathic pulmonary fibrosis"[MeSH Terms] OR "idiopathic pulmonary fibrosis"[tiab] OR IPF[tiab])) AND ("2000/01/01"[Date - Publication] : "2026/12/31"[Date - Publication])

PubMed focused PH-ILD search (87 records)

(("treprostinil"[MeSH Terms] OR treprostinil[tiab] OR "inhaled treprostinil"[tiab] OR Tyvaso[tiab]) AND ("interstitial lung disease"[MeSH Terms] OR "interstitial lung disease"[tiab] OR "interstitial lung diseases"[tiab] OR ILD[tiab]) AND ("pulmonary hypertension"[MeSH Terms] OR "pulmonary hypertension"[tiab] OR "PH-ILD"[tiab] OR "group 3 pulmonary hypertension"[tiab])) AND ("2000/01/01"[Date - Publication] : "2026/12/31"[Date - Publication])

Google Scholar main search (913 records)

"inhaled treprostinil" "idiopathic pulmonary fibrosis"

Google Scholar PH-ILD verification search (1370 records)

"inhaled treprostinil" "pulmonary hypertension" "interstitial lung disease"

Google Scholar TETON verification search (161 records)

treprostinil TETON idiopathic pulmonary fibrosis

Google Scholar INCREASE FVC verification search (1300 records)

treprostinil INCREASE forced vital capacity interstitial lung disease pulmonary hypertension

Cochrane Search 1 - main broad search (190 records: 188 trials and 2 reviews)

(treprostinil OR "inhaled treprostinil" OR Tyvaso OR "Tyvaso DPI") AND ("idiopathic pulmonary fibrosis" OR IPF OR "interstitial lung disease" OR "interstitial lung diseases" OR ILD OR "pulmonary fibrosis" OR "progressive pulmonary fibrosis" OR "pulmonary hypertension" OR PH-ILD OR "pulmonary hypertension associated with interstitial lung disease" OR "group 3 pulmonary hypertension")

Cochrane Search 2 - focused IPF search (18 trials)

(treprostinil OR "inhaled treprostinil" OR Tyvaso OR "Tyvaso DPI") AND ("idiopathic pulmonary fibrosis" OR IPF)

Cochrane Search 3 - focused PH-ILD / INCREASE search (57 trials)

(treprostinil OR "inhaled treprostinil" OR Tyvaso OR "Tyvaso DPI") AND ("interstitial lung disease" OR "interstitial lung diseases" OR ILD) AND ("pulmonary hypertension" OR PH-ILD OR "group 3 pulmonary hypertension")

Google Scholar searches used the custom year range 2000-2026 and were used as a broad supplementary identification source within the search strategy. Because Google Scholar ranking is dynamic and its results are not fully reproducible, this is acknowledged as a search-method limitation. No additional Cochrane date syntax was embedded in the supplied search strings; the prespecified review eligibility window was applied during study selection. Embase was not searched because access was unavailable, which is also acknowledged as a limitation.

Supplementary Methods S1B. Protocol chronology, registration and review procedures

The review protocol, including eligibility criteria, outcomes and the planned analytical approach, was developed and finalized before study screening commenced. The protocol was not prospectively registered and was subsequently registered retrospectively in PROSPERO (registration number: CRD420261497663). Retrospective registration is reported transparently as a methodological limitation.

Title and abstract screening and full-text eligibility assessment were performed independently by the first and fourth authors. Disagreements were recorded and resolved through adjudication meetings involving all authors until consensus was reached. Data extraction was divided equally between the first and second authors. Each author independently cross-checked the other author's extraction against the original source publication before finalization, and discrepancies were resolved by discussion.

Supplementary Table S2. PRISMA flow counts and exclusion reasons

PRISMA item	Count
Records identified from databases	1552
Duplicates removed	1173
Records after deduplication	379
Records excluded after title and abstract screening	294
Full-text reports assessed	85
Full-text reports excluded	74
Wrong study design	44
Wrong intervention or exposure	13
Ineligible comparison group or PICO mismatch	17
Reports included in systematic review	11
Clinical outcome-data reports	10
Direct IPF randomized trials in primary meta-analysis	2

Supplementary Table S3. Included reports and pooling role

Study	Region	Design	Population	N	Arms	Follow-up	Role
TETON-1	United States and Canada	Phase 3 double-blind RCT	Direct IPF	598	299/299	52 weeks	Main meta-analysis

Study	Region	Design	Population	N	Arms	Follow-up	Role
TETON-2	Asia-Pacific, Europe and South America	Phase 3 double-blind RCT	Direct IPF	593	298/295	52 weeks	Main meta-analysis
INCREASE	United States	Phase 3 double-blind RCT	PH-ILD; IPF subgroup n = 92	326	163/163	16 weeks	Secondary and sensitivity
INCREASE FVC post hoc	United States	Post hoc RCT analysis	PH-ILD; IPF subgroup n = 92	326	163/163	16 weeks	Secondary and narrative
INCREASE disease progression	United States	Post hoc RCT analysis	PH-ILD	326	163/163	16 weeks	Narrative only
INCREASE dosage	United States	Post hoc RCT analysis	PH-ILD	302	149/153	16 weeks	Narrative only
INCREASE OLE	United States	Open-label extension	PH-ILD	242	242/NA	Median exposure 62.1 weeks	Narrative only
INCREASE less severe hemodynamics	United States and Canada	Post hoc RCT analysis	PH-ILD	326	163/163	16 weeks; OLE to 1 year	Narrative only
Japanese PH-ILD study	Japan	Prospective single-arm study	PH-ILD; IPF subgroup n = 7	20	20/NA	52 weeks	Narrative only
Japanese long-term follow-up	Japan	Open-label long-term follow-up	PH-ILD	20	20/NA	Median 107.5 weeks	Narrative only
TETON design paper	International	Trial protocol/design	Direct IPF trial design	NA	NA	NA	Design background only

Supplementary Table S4. Baseline and intervention extraction

Dataset	Mean age	Male	Baseline FVC	Baseline DLCO	Background antifibrotic use	Intervention
TETON-1	73.0	77.3%	74.6%	NR	77.6%	Three breaths [18 micrograms] four times daily, titrated to 12 breaths [72 micrograms] four times daily
TETON-2	71.7	80.1%	76.8%	NR	75.4%	Same as TETON-1
INCREASE	66.5	54.0%	62.5%	NR	Variable background therapy	Three breaths four times daily, titrated to 9 to 12 breaths four times daily
Japanese PH-ILD study	NR	NR	NR	NR	NR	Inhaled treprostinil, titrated to tolerated dose

Supplementary Table S5. Main efficacy extraction from direct IPF randomized trials

Study	Outcome	Effect	95% CI	p-value	Effect type
TETON-1	Absolute FVC change at 52 weeks	130.1 mL	82.2 to 178.1	<0.001	Hodges–Lehmann location shift
TETON-2	Absolute FVC change at 52 weeks	95.6 mL	52.2 to 139.0	<0.001	Hodges–Lehmann location shift
TETON-1	% predicted FVC change at 52 weeks	3.7%	2.4 to 5.0	NR	Hodges–Lehmann location shift
TETON-2	% predicted FVC change at 52 weeks	2.7%	1.5 to 4.0	NR	Hodges–Lehmann location shift
TETON-1	K-BILD total score change at 52 weeks	1.4	-0.2 to 2.9	NR	Hodges–Lehmann location shift
TETON-2	K-BILD total score change at 52 weeks	2.3	0.5 to 4.1	NR	Hodges–Lehmann location shift
TETON-1	% predicted DLCO change at 52 weeks	1.9%	-0.2 to 3.9	NR	Hodges–Lehmann location shift
TETON-2	% predicted DLCO change at 52 weeks	1.9%	0.1 to 3.8	NR	Hodges–Lehmann location shift

Supplementary Table S6. Time-to-event extraction from direct IPF randomized trials

Study	Outcome	Treprostinil events/N	Placebo events/N	HR	95% CI	p-value
TETON-1	Clinical worsening	95/299	133/299	0.67	0.52 to 0.88	0.003
TETON-2	Clinical worsening	81/298	115/295	0.71	0.53 to 0.95	0.02
TETON-1	Acute exacerbation of IPF	10/299	19/299	0.50	0.23 to 1.08	NR
TETON-2	Acute exacerbation of IPF	9/298	17/295	0.54	0.24 to 1.22	NR
TETON-1	All-cause mortality	22/299	32/299	0.65	0.38 to 1.13	NR
TETON-2	All-cause mortality	19/298	24/295	0.77	0.42 to 1.41	NR

Supplementary Table S7. Safety extraction from direct IPF randomized trials

Study	Safety outcome	Treprostinil	Placebo
TETON-1	Any AE	287/299	280/299
TETON-1	Serious AE	54/299	72/299
TETON-1	AE leading to discontinuation	62/299	44/299
TETON-1	Death	22/299	32/299

Study	Safety outcome	Treprostinil	Placebo
TETON-1	Cough	164/299	99/299
TETON-1	Headache	91/299	48/299
TETON-1	Dyspnea	56/299	47/299
TETON-1	Diarrhea	49/299	63/299
TETON-2	Any AE	273/298	264/295
TETON-2	Serious AE	75/298	68/295
TETON-2	AE leading to discontinuation	50/298	37/295
TETON-2	Death	19/298	24/295
TETON-2	Cough	144/298	71/295
TETON-2	Headache	59/298	35/295
TETON-2	Dyspnea	36/298	31/295
TETON-2	Diarrhea	41/298	50/295
TETON-2	Dizziness	29/298	19/295
TETON-2	Nausea	24/298	26/295
TETON-2	Throat irritation or pharyngolaryngeal pain	21/298	7/295

Supplementary Table S8. Secondary and narrative datasets

Dataset	Outcome	Effect	95% CI	p-value	Role
INCREASE IPF subgroup	Absolute FVC at week 16	168.5 mL	40.1 to 297.0	0.011	Secondary sensitivity only
INCREASE IPF subgroup	% predicted FVC at week 16	3.5%	0.7 to 6.3	0.015	Secondary sensitivity only
INCREASE overall PH-ILD	6MWD at week 16	31.12 m	16.85 to 45.39	<0.001	PH-ILD evidence only
INCREASE disease progression	Time to first progression event	HR 0.71	0.54 to 0.94	0.019	Narrative only
INCREASE dosage	Clinical worsening from weeks 4 to 16	17.1% in high-dose treprostinil vs about 34% in placebo groups	NR	0.006	Narrative only
INCREASE less severe hemodynamics	Exacerbations in PVR <=5 WU subgroup	HR 0.43	0.23 to 0.81	0.01	Narrative only
INCREASE OLE	6MWD change from RCT baseline to OLE week 52	3.5 m	NR	NR	Narrative only
Japanese PH-ILD study	PVRI change at week 16	-40.1%	-53.1 to -27.2	NR	Narrative only
Japanese long-term follow-up	Death- or transplant-free survival	100.0% at 1 year, 93.8% at 2 years and 75.0% at 3 years	NR	NR	Narrative only

Supplementary Table S9. Risk of bias and quality assessment

Dataset or analysis	Tool	Judgement	Main reason
TETON-1	RoB 2	Some concerns	High attrition before week 52 and incomplete sequence details in the main text; central spirometry and blinded event adjudication supported outcome measurement.
TETON-2	RoB 2	Some concerns	High attrition before week 52 and incomplete sequence details in the main text; sensitivity analyses supported the primary result.
INCREASE	RoB 2	Some concerns	Central randomization and double-blinding were used; clinical worsening and exacerbations were investigator-reported without independent adjudication.
INCREASE post hoc analyses	Methodological critique	Exploratory	Shared parent dataset, retrospective subgrouping, no multiplicity control and some post-randomization grouping.
INCREASE OLE	Adapted NOS	Low to moderate	Single-arm open-label design, no concurrent comparator and high discontinuation.
Japanese PH-ILD studies	Adapted NOS	Low to moderate	Small single-arm cohort, no comparator and survivor effects during long-term follow-up.

Supplementary Table S10. Pooled random-effects results

Forest plot index	Outcome	Measure	Pooled effect	95% CI	p-value	I squared	tau squared	Interpretation
1	Absolute FVC change	MD	111.28 mL	77.61 to 144.95	<0.001	8.5%	50.7213	Favors treprostinil
2	% predicted FVC change	MD	3.18%	2.20 to 4.16	<0.001	15.3%	0.0767	Favors treprostinil
3	K-BILD total score	MD	1.78	0.61 to 2.96	0.003	0.0%	0.00	Favors treprostinil
4	% predicted DLCO change	MD	1.90%	0.53 to 3.27	0.007	0.0%	0.00	Favors treprostinil
5	Clinical worsening	HR	0.69	0.57 to 0.84	<0.001	0.0%	0.00	Favors treprostinil
6	Acute exacerbation of IPF	HR	0.52	0.30 to 0.91	0.022	0.0%	0.00	Favors treprostinil

Forest plot index	Outcome	Measure	Pooled effect	95% CI	p-value	I squared	tau squared	Interpretation
7	All-cause mortality	HR	0.70	0.47 to 1.05	0.086	0.0%	0.00	Not significant
8	Any adverse event	RR	1.02	0.99 to 1.06	0.118	0.0%	0.00	Not significant
9	Serious adverse event	RR	0.91	0.63 to 1.31	0.616	66.7%	0.0470	Not significant
10	AE leading to discontinuation	RR	1.38	1.06 to 1.79	0.017	0.0%	0.00	Higher with treprostinil
11	Cough	RR	1.80	1.49 to 2.17	<0.001	35.6%	0.0066	Higher with treprostinil
12	Headache	RR	1.80	1.42 to 2.30	<0.001	0.0%	0.00	Higher with treprostinil
13	Dyspnea	RR	1.18	0.89 to 1.55	0.255	0.0%	0.00	Not significant
14	Diarrhea	RR	0.79	0.62 to 1.02	0.071	0.0%	0.00	Not significant

Supplementary Table S11. Effect measure definitions and interpretation rules

The review used MD for continuous efficacy outcomes, HR for time-to-event outcomes and RR for safety outcomes. OR and SMD were not used. The null value is 0 for MD and 1 for HR, RR and OR. A result was considered statistically significant when the 95% CI did not include the null value.

Supplementary Table S11. Effect measure definitions and interpretation rules

Effect measure	Used	Applied to	Format	Null	Interpretation
Mean Difference (MD)	Yes	Absolute FVC, % predicted FVC, K-BILD, % predicted DLCO	MD = estimate (95% CI lower to upper)	0	Positive values favor inhaled treprostinil for efficacy outcomes. Significant when the 95% CI does not include 0.
Standardized Mean Difference (SMD)	No	Not required because outcomes used the same scale across trials	SMD = estimate (95% CI lower to upper)	0	Not applied.
Hazard Ratio (HR)	Yes	Clinical worsening, acute exacerbation, all-cause mortality	HR = estimate (95% CI lower to upper)	1	HR below 1 favors inhaled treprostinil. Relative hazard reduction = (1 - HR) x 100. Significant when the 95% CI does not include 1.
Risk Ratio (RR)	Yes	Safety outcomes from raw event counts	RR = estimate (95% CI lower to upper)	1	RR below 1 indicates lower event risk with treprostinil. RR above 1 indicates higher event risk. Relative change = absolute distance from 1 x 100. Significant when the 95% CI does not include 1.
Odds Ratio (OR)	No	Not used for pooled safety analyses	OR = estimate (95% CI lower to upper)	1	Not applied because RR was more directly interpretable for safety event counts.

Supplementary Table S12. Outcome-specific effect measures and statistical interpretation

This table converts the pooled forest plot results into the requested format and identifies whether each 95% CI crossed the null value. Relative reduction or increase is not calculated for MD because those outcomes use their original measurement units.

Supplementary Table S12. Outcome-specific effect measures and statistical interpretation

Outcome	Measure	Pooled result format	CI crosses null?	Statistical interpretation	Relative interpretation
Absolute FVC change	MD	MD = 111.28 mL (95% CI 77.61 to 144.95)	No	Significant; favors inhaled treprostinil	Absolute benefit was 111.28 mL
% predicted FVC change	MD	MD = 3.18% (95% CI 2.20 to 4.16)	No	Significant; favors inhaled treprostinil	Not applicable for MD
K-BILD total score change	MD	MD = 1.78 (95% CI 0.61 to 2.96)	No	Significant pooled estimate; nominal interpretation because of trial hierarchy	Not applicable for MD
% predicted DLCO change	MD	MD = 1.90% (95% CI 0.53 to 3.27)	No	Significant pooled estimate; nominal interpretation because of trial hierarchy	Not applicable for MD
Clinical worsening	HR	HR = 0.69 (95% CI 0.57 to 0.84)	No	Significant; favors inhaled treprostinil	31% relative hazard reduction
Acute exacerbation of IPF	HR	HR = 0.52 (95% CI 0.30 to 0.91)	No	Significant; favors inhaled treprostinil in pooled analysis	48% relative hazard reduction
All-cause mortality	HR	HR = 0.70 (95% CI 0.47 to 1.05)	Yes	Not significant	Estimated 30% relative hazard reduction; not statistically significant
Any adverse event	RR	RR = 1.02 (95% CI 0.99 to 1.06)	Yes	Not significant	Estimated 2% relative risk increase; not statistically significant
Serious adverse event	RR	RR = 0.91 (95% CI 0.63 to 1.31)	Yes	Not significant	Estimated 9% relative risk reduction; not statistically significant
AE leading to discontinuation	RR	RR = 1.38 (95% CI 1.06 to 1.79)	No	Significant; higher with inhaled treprostinil	38% relative risk increase
Cough	RR	RR = 1.80 (95% CI 1.49 to 2.17)	No	Significant; higher with inhaled treprostinil	80% relative risk increase
Headache	RR	RR = 1.80 (95% CI 1.42 to 2.30)	No	Significant; higher with inhaled treprostinil	80% relative risk increase
Dyspnea	RR	RR = 1.18 (95% CI 0.89 to 1.55)	Yes	Not significant	Estimated 18% relative risk increase; not statistically significant

Outcome	Measure	Pooled result format	CI crosses null?	Statistical interpretation	Relative interpretation
Diarrhea	RR	RR = 0.79 (95% CI 0.62 to 1.02)	Yes	Not significant	Estimated 21% relative risk reduction; not statistically significant

Supplementary Methods S3. Two-stage synthesis and statistical sensitivity analyses

For continuous and time-to-event outcomes, the meta-analysis used a two-stage aggregate-data approach. Stage 1 used each trial's reported treatment effect and variance. For continuous outcomes, standard errors were derived from 95% confidence intervals as (upper limit - lower limit)/(2 x 1.96). For hazard ratios, standard errors were derived on the log scale as [ln(upper limit) - ln(lower limit)]/(2 x 1.96). For safety risk ratios derived from event counts, log-risk-ratio variance used 1/a - 1/nT + 1/c - 1/nC, where a and c are events and nT and nC are randomized/safety denominators. Stage 2 pooled the trial-level estimates by inverse variance. No standardized mean difference or odds ratio was used.

Because k = 2 for each direct-IPF pooled analysis, the primary DerSimonian–Laird model was compared with fixed-effect inverse variance, REML random effects and modified Hartung–Knapp inference using REML. The modified Hartung–Knapp scale factor was bounded below at 1 and inference used a t distribution with k - 1 degrees of freedom. This was treated as a conservative small-k sensitivity analysis. Conventional leave-one-out pooling was not performed because removing either trial leaves one study.

Supplementary Table S13. Statistical sensitivity analyses for all pooled outcomes.

Outcome	DL random effects	Fixed effect	REML random effects	Modified Hartung–Knapp	Point-estimate direction
Absolute FVC change	MD 111.28 (77.61 to 144.95)	MD 111.14 (78.96 to 143.31)	MD 111.28 (77.61 to 144.95)	MD 111.28 (-106.99 to 329.56)	Yes
% predicted FVC	MD 3.18 (2.20 to 4.16)	MD 3.18 (2.28 to 4.08)	MD 3.18 (2.20 to 4.16)	MD 3.18 (-3.17 to 9.53)	Yes
K-BILD total score	MD 1.78 (0.61 to 2.96)	MD 1.78 (0.61 to 2.96)	MD 1.78 (0.61 to 2.96)	MD 1.78 (-5.83 to 9.40)	Yes
% predicted DLCO	MD 1.90 (0.53 to 3.27)	MD 1.90 (0.53 to 3.27)	MD 1.90 (0.53 to 3.27)	MD 1.90 (-7.00 to 10.80)	Yes
Clinical worsening	HR 0.69 (0.57 to 0.84)	HR 0.69 (0.57 to 0.84)	HR 0.69 (0.57 to 0.84)	HR 0.69 (0.19 to 2.44)	Yes
Acute exacerbation	HR 0.52 (0.30 to 0.91)	HR 0.52 (0.30 to 0.91)	HR 0.52 (0.30 to 0.91)	HR 0.52 (0.01 to 19.61)	Yes
All-cause mortality	HR 0.70 (0.47 to 1.05)	HR 0.70 (0.47 to 1.05)	HR 0.70 (0.47 to 1.05)	HR 0.70 (0.05 to 9.69)	Yes
Any adverse event	RR 1.02 (0.99 to 1.06)	RR 1.02 (0.99 to 1.06)	RR 1.02 (0.99 to 1.06)	RR 1.02 (0.84 to 1.25)	Yes
Serious adverse event	RR 0.91 (0.63 to 1.31)	RR 0.92 (0.75 to 1.14)	RR 0.91 (0.63 to 1.31)	RR 0.91 (0.08 to 9.88)	Direction not uniform across trials
AE leading to discontinuation	RR 1.38 (1.06 to 1.79)	RR 1.38 (1.06 to 1.79)	RR 1.38 (1.06 to 1.79)	RR 1.38 (0.25 to 7.53)	Yes
Cough	RR 1.80 (1.49 to 2.17)	RR 1.79 (1.54 to 2.07)	RR 1.80 (1.49 to 2.17)	RR 1.80 (0.54 to 6.05)	Yes
Headache	RR 1.80 (1.42 to 2.30)	RR 1.80 (1.42 to 2.30)	RR 1.80 (1.42 to 2.30)	RR 1.80 (0.38 to 8.66)	Yes
Dyspnea	RR 1.18 (0.89 to 1.55)	RR 1.18 (0.89 to 1.55)	RR 1.18 (0.89 to 1.55)	RR 1.18 (0.19 to 7.14)	Yes
Diarrhea	RR 0.79 (0.62 to 1.02)	RR 0.79 (0.62 to 1.02)	RR 0.79 (0.62 to 1.02)	RR 0.79 (0.15 to 4.07)	Yes

Supplementary Methods S4. GRADE certainty-of-evidence assessment

Certainty of evidence for major direct-IPF outcomes was rated using GRADE [46]. Randomized evidence started at high certainty and was assessed for risk of bias, inconsistency, indirectness, imprecision and publication bias. The ratings below apply only to TETON-1 and TETON-2. INCREASE and other PH-ILD evidence were excluded from these direct-IPF certainty ratings because of population and follow-up indirectness.

Supplementary Table S14. GRADE evidence profile for major direct-IPF outcomes.

Outcome	Studies / participants	Pooled effect	GRADE domains	Certainty	Reason for rating
Absolute FVC change at 52 weeks	2 RCTs; 1191	MD 111.28 mL (77.61 to 144.95)	RoB: not serious; inconsistency: not serious; indirectness: not serious; imprecision: serious; publication bias: not detected	Moderate	Downgraded one level for imprecision because the CI spans values below and above a commonly cited ~100 mL clinical threshold and small-k adjusted inference was wide.
Clinical worsening at 52 weeks	2 RCTs; 1191	HR 0.69 (0.57 to 0.84)	RoB: not serious; inconsistency: not serious; indirectness: not serious; imprecision: serious; publication bias: not detected	Moderate	Downgraded one level for imprecision because only two trials inform between-study uncertainty and the modified Hartung–Knapp interval crossed the null.
Acute exacerbation of IPF	2 RCTs; 1191	HR 0.52 (0.30 to 0.91)	RoB: not serious; inconsistency: not serious; indirectness: not serious; imprecision: very serious; publication bias: not detected	Low	Downgraded two levels for very serious imprecision because only 55 events occurred and small-k adjusted inference was extremely wide.
All-cause mortality	2 RCTs; 1191	HR 0.70 (0.47 to 1.05)	RoB: not serious; inconsistency: not serious; indirectness: not serious; imprecision: very serious; publication bias: not detected	Low	Downgraded two levels for very serious imprecision because only 97 deaths occurred and the CI includes both no effect and a clinically important reduction in mortality.
Serious adverse events	2 RCTs; 1191	RR 0.91 (0.63 to 1.31)	RoB: not serious; inconsistency: serious; indirectness: not serious; imprecision: serious; publication bias: not detected	Low	Downgraded for inconsistency (I squared 66.7% with different trial-specific directions) and imprecision because the CI includes potentially important benefit and harm.

Outcome	Studies / participants	Pooled effect	GRADE domains	Certainty	Reason for rating
AE leading to discontinuation	2 RCTs; 1191	RR 1.38 (1.06 to 1.79)	RoB: not serious; inconsistency: not serious; indirectness: not serious; imprecision: serious; publication bias: not detected	Moderate	Downgraded one level for imprecision because the lower confidence limit is close to the null and small-k adjusted inference was wide.

GRADE note: RoB 2 identified some concerns in the direct IPF trials, chiefly attrition and incomplete reporting of sequence-generation details. The GRADE risk-of-bias domain was not downgraded because central spirometry, double blinding, treatment-policy estimands, prespecified imputation and supportive primary-endpoint sensitivity analyses were judged to reduce the likelihood that these concerns materially changed the main effect estimates. This does not reclassify the RoB 2 judgement as low risk. Formal publication-bias testing was not informative with only two direct trials.

Supplementary Methods S2. Double-counting and pooling rules

Dataset	Reason	Analysis decision	Bias avoided
TETON-1	Separately randomized direct IPF trial within coordinated TETON program	Included in primary pooling	Only one row per outcome
TETON-2	Separately randomized direct IPF trial within coordinated TETON program	Included in primary pooling	Only one row per outcome
Combined TETON analysis	Prespecified combined analysis of TETON-1 and TETON-2 from the same coordinated program	Excluded from pooling; used only as supportive context	Supportive only to avoid double-counting
INCREASE parent trial	PH-ILD RCT	Not pooled with direct IPF trials	Different population and 16-week follow-up
INCREASE FVC post hoc	Same INCREASE dataset	Secondary sensitivity only	IPF subgroup was post hoc and shorter follow-up
INCREASE other post hoc papers	Same INCREASE dataset	Narrative only	Shared cohort and exploratory analyses
INCREASE OLE	Open-label extension	Narrative only	No concurrent comparator
Japanese PH-ILD studies	Same single-arm Japanese cohort	Narrative only	Small uncontrolled cohort

Supplementary Note S1. Reference and citation integrity exit criteria

Rule	Exit criterion	Draft status	Evidence or action
V01	Every included report appears in the reference list	Pass in draft	All 11 included reports are cited.
V02	Only TETON-1 and TETON-2 are marked as main meta-analysis datasets	Pass	All other datasets are labeled secondary or narrative.

Rule	Exit criterion	Draft status	Evidence or action
V03	Combined TETON data are not pooled with individual TETON trials	Pass	Combined TETON is not used in any forest plot.
V04	Every reference in the reference list has at least one in-text citation	Pass in draft	Main manuscript was assembled with sequential citation use.
V05	Reference numbering follows first appearance across text, tables and figures	Pass in draft	References were written in manuscript-order numbering.
V06	All effect estimates, p-values, confidence intervals and sample sizes match extracted data	Pass in draft	Numbers were copied from extraction tables and forest plot summary.
V07	Rows without confirmed PMID are not falsely labeled as PubMed validated	Pass with caution	DOI-verified included studies without PMID are retained and should be updated when PubMed entries are available.
V08	Exact database-specific search strings and final run date are reproduced in the supplement	Pass	PubMed, Google Scholar and Cochrane strategies documented; final search run 3 July 2026.
V09	Primary PRISMA count excludes focused verification searches	Pass	PRISMA identification total remains 1552 from the three main searches.
V10	Small-k sensitivity analyses are reported for all 14 pooled outcomes	Pass	DL, fixed effect, REML and modified Hartung–Knapp results are in Supplementary Table S13.
V11	GRADE is completed for major direct-IPF outcomes	Pass	Domain-level judgments and reasons for downgrading are in Supplementary Table S14.

Supplementary Figure S1. Forest plot figure index

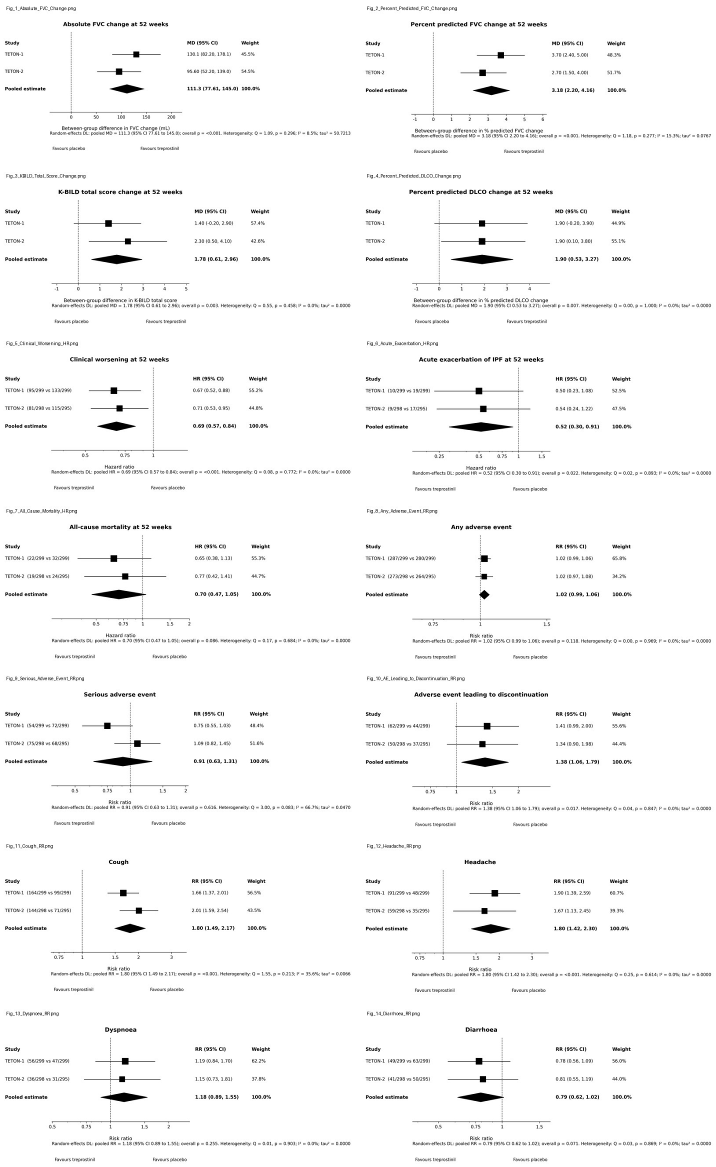

Supplementary Figure S1. Contact sheet of all 14 forest plots used in the manuscript.

Supplementary References

1. Raghu G, Remy-Jardin M, Myers JL, et al. Diagnosis of idiopathic pulmonary fibrosis. An official ATS/ERS/JRS/ALAT clinical practice guideline. Am J Respir Crit Care Med. 2018;198:e44-e68. doi:10.1164/rccm.201807-1255ST.
2. Raghu G, Remy-Jardin M, Richeldi L, et al. Idiopathic pulmonary fibrosis and progressive pulmonary fibrosis in adults. An official ATS/ERS/JRS/ALAT clinical practice guideline. Am J Respir Crit Care Med. 2022;205:e18-e47. doi:10.1164/rccm.202202-0399ST.
3. Lynch DA, Sverzellati N, Travis WD, et al. Diagnostic criteria for idiopathic pulmonary fibrosis: a Fleischner Society white paper. Lancet Respir Med. 2018;6:138-153. doi:10.1016/S2213-2600(17)30433-2.
4. Martinez FJ, Safrin S, Weycker D, et al. The clinical course of patients with idiopathic pulmonary fibrosis. Ann Intern Med. 2005;142:963-967.
5. Ley B, Collard HR, King TE Jr. Clinical course and prediction of survival in idiopathic pulmonary fibrosis. Am J Respir Crit Care Med. 2011;183:431-440. doi:10.1164/rccm.201006-0894CI.
6. Collard HR, King TE Jr, Bartelson BB, Vourlekis JS, Schwarz MI, Brown KK. Changes in clinical and physiologic variables predict survival in idiopathic pulmonary fibrosis. Am J Respir Crit Care Med. 2003;168:538-542. doi:10.1164/rccm.200211-1311OC.
7. Paterniti MO, Bi Y, Rekic D, Wang Y, Karimi-Shah BA, Chowdhury BA. Acute exacerbation and decline in forced vital capacity are associated with increased mortality in idiopathic pulmonary fibrosis. Ann Am Thorac Soc. 2017;14:1395-1402. doi:10.1513/AnnalsATS.201702-140OC.
8. Durheim MT, Collard HR, Roberts RS, et al. Association of hospital admission and forced vital capacity endpoints with survival in patients with idiopathic pulmonary fibrosis: analysis of a pooled cohort from three clinical trials. Lancet Respir Med. 2015;3:388-396. doi:10.1016/S2213-2600(15)00099-4.
9. Brown AW, Fischer CP, Shlobin OA, et al. Outcomes after hospitalization in idiopathic pulmonary fibrosis: a cohort study. Chest. 2015;147:173-179. doi:10.1378/chest.13-2424.
10. Noble PW, Albera C, Bradford WZ, et al. Pirfenidone in patients with idiopathic pulmonary fibrosis (CAPACITY): two randomised trials. Lancet. 2011;377:1760-1769. doi:10.1016/S0140-6736(11)60405-4.
11. King TE Jr, Bradford WZ, Castro-Bernardini S, et al. A phase 3 trial of pirfenidone in patients with idiopathic pulmonary fibrosis. N Engl J Med. 2014;370:2083-2092. doi:10.1056/NEJMoa1402582.
12. Richeldi L, du Bois RM, Raghu G, et al. Efficacy and safety of nintedanib in idiopathic pulmonary fibrosis. N Engl J Med. 2014;370:2071-2082. doi:10.1056/NEJMoa1402584.
13. Maher TM, Molina-Molina M, Russell AM, et al. Unmet needs in the treatment of idiopathic pulmonary fibrosis: insights from patient chart review in five European countries. BMC Pulm Med. 2017;17:124. doi:10.1186/s12890-017-0468-5.
14. Dempsey TM, Payne S, Sangaralingham L, Yao X, Shah ND, Limper AH. Adoption of the antifibrotic medications pirfenidone and nintedanib for patients with idiopathic pulmonary fibrosis. Ann Am Thorac Soc. 2021;18:1121-1128. doi:10.1513/AnnalsATS.202007-901OC.
15. Humbert M, Kovacs G, Hoeper MM, et al. 2022 ESC/ERS Guidelines for the diagnosis and treatment of pulmonary hypertension. Eur Heart J. 2022;43:3618-3731. doi:10.1093/eurheartj/ehac237.
16. Simonneau G, Montani D, Celermajer DS, et al. Haemodynamic definitions and updated clinical classification of pulmonary hypertension. Eur Respir J. 2019;53:1801913. doi:10.1183/13993003.01913-2018.
17. Nathan SD, Barbera JA, Gaine SP, et al. Pulmonary hypertension in chronic lung disease and hypoxia. Eur Respir J. 2019;53:1801914. doi:10.1183/13993003.01914-2018.
18. Nathan SD. Pulmonary hypertension in interstitial lung disease. Int J Clin Pract Suppl. 2008;(160):21-28. doi:10.1111/j.1742-1241.2008.01810.x.
19. Nathan SD, Behr J, Collard HR, et al. Riociguat for idiopathic interstitial pneumonia-associated pulmonary hypertension (RISE-IIP): a randomised, placebo-controlled phase 2b study. Lancet Respir Med. 2019;7:780-790. doi:10.1016/S2213-2600(19)30156-8.
20. Corte TJ, Keir GJ, Dimopoulos K, et al. Bosentan in pulmonary hypertension associated with fibrotic idiopathic interstitial pneumonia. Am J Respir Crit Care Med. 2014;190:208-217. doi:10.1164/rccm.201403-0446OC.
21. McLaughlin VV, Benza RL, Rubin LJ, et al. Addition of inhaled treprostinil to oral therapy for pulmonary arterial hypertension: a randomized controlled clinical trial. J Am Coll Cardiol. 2010;55:1915-1922. doi:10.1016/j.jacc.2010.01.027.
22. Lambers C, Roth M, Jaksch P, et al. Treprostinil inhibits proliferation and extracellular matrix deposition by fibroblasts through cAMP activation. Sci Rep. 2018;8:1087. doi:10.1038/s41598-018-19295-2.
23. Zmajkovicova K, Menyhart K, Bauer Y, et al. The antifibrotic activity of prostacyclin receptor agonism is mediated through inhibition of YAP/TAZ. Am J Respir Cell Mol Biol. 2019;60:578-591. doi:10.1165/rcmb.2018-0301OC.

24. Roberts MJ, May LT, Keen AC, et al. Inhibition of the proliferation of human lung fibroblasts by prostacyclin receptor agonists is linked to a sustained cAMP signal in the nucleus. Front Pharmacol. 2021;12:669227. doi:10.3389/fphar.2021.669227.
25. Nikitopoulou I, Manitsopoulos N, Kotanidou A, et al. Orotracheal treprostinil administration attenuates bleomycin-induced lung injury, vascular remodeling, and fibrosis in mice. Pulm Circ. 2019;9:2045894019871430. doi:10.1177/2045894019871430.
26. Waxman A, Restrepo-Jaramillo R, Thenappan T, et al. Inhaled treprostinil in pulmonary hypertension due to interstitial lung disease. N Engl J Med. 2021;384:325-334. doi:10.1056/NEJMoa2008470.
27. Nathan SD, Waxman A, Rajagopal S, et al. Inhaled treprostinil and forced vital capacity in patients with interstitial lung disease and associated pulmonary hypertension: a post-hoc analysis of the INCREASE study. Lancet Respir Med. 2021;9:1266-1274. doi:10.1016/S2213-2600(21)00165-X.
28. Nathan SD, Behr J, Cottin V, et al. Study design and rationale for the TETON phase 3, randomised, controlled clinical trials of inhaled treprostinil in the treatment of idiopathic pulmonary fibrosis. BMJ Open Respir Res. 2022;9:e001310. doi:10.1136/bmjresp-2022-001310.
29. Nathan SD, Smith P, Deng C, et al. Phase 3 trials of inhaled treprostinil for idiopathic pulmonary fibrosis. N Engl J Med. 2026;395:115-126. doi:10.1056/NEJMoa2501488.
30. Nathan SD, Smith P, Deng C, et al. Inhaled treprostinil for idiopathic pulmonary fibrosis. N Engl J Med. 2026;395:127-137. doi:10.1056/NEJMoa2512911.
31. Page MJ, McKenzie JE, Bossuyt PM, et al. The PRISMA 2020 statement: an updated guideline for reporting systematic reviews. BMJ. 2021;372:n71. doi:10.1136/bmj.n71.
32. DerSimonian R, Laird N. Meta-analysis in clinical trials. Control Clin Trials. 1986;7:177-188. doi:10.1016/0197-2456(86)90046-2.
33. Higgins JP, Thompson SG, Deeks JJ, Altman DG. Measuring inconsistency in meta-analyses. BMJ. 2003;327:557-560. doi:10.1136/bmj.327.7414.557.
34. du Bois RM, Weycker D, Albera C, et al. Forced vital capacity in patients with idiopathic pulmonary fibrosis: test properties and minimal clinically important difference. Am J Respir Crit Care Med. 2011;184:1382-1389. doi:10.1164/rccm.201105-0840OC.
35. Prior TS, Hoyer N, Hilberg O, Shaker SB, Davidsen JR, Bendstrup E. Responsiveness and minimal clinically important difference of SGRQ-I and K-BILD in idiopathic pulmonary fibrosis. Respir Res. 2020;21:91. doi:10.1186/s12931-020-01352-3.
36. Nathan SD, du Bois RM, Albera C, et al. Validation of test performance characteristics and minimal clinically important difference of the 6-minute walk test in patients with idiopathic pulmonary fibrosis. Respir Med. 2015;109:914-922. doi:10.1016/j.rmed.2015.04.008.
37. Nathan SD, Tapson VF, Elwing J, et al. Efficacy of inhaled treprostinil on multiple disease progression events in patients with pulmonary hypertension due to parenchymal lung disease in the INCREASE trial. Am J Respir Crit Care Med. 2022;205:198-207. doi:10.1164/rccm.202107-1764OC.
38. Nathan SD, Deng C, Chen J, et al. Treatment response and safety of inhaled treprostinil in patients with pulmonary hypertension due to interstitial lung disease based on dose. Chest. 2023;163:398-406. doi:10.1016/j.chest.2022.09.007.
39. Waxman A, Restrepo-Jaramillo R, Thenappan T, et al. Long-term inhaled treprostinil for pulmonary hypertension due to interstitial lung disease: INCREASE open-label extension study. Eur Respir J. 2023;61:2202414. doi:10.1183/13993003.02414-2022.
40. Weatherald J, et al. Inhaled treprostinil in patients with pulmonary hypertension associated with interstitial lung disease and less severe haemodynamics: a post-hoc analysis. BMJ Open Respir Res. 2024;11:e002116. doi:10.1136/bmjresp-2023-002116.
41. Sakao S, et al. Efficacy, safety, and pharmacokinetics of inhaled treprostinil in Japanese patients with pulmonary hypertension associated with interstitial lung disease. Respir Investig. 2024;62:980-986.
42. Kondoh Y, et al. Long-term effects of inhaled treprostinil in Japanese patients with pulmonary hypertension associated with interstitial lung disease. Respir Investig. 2025.
43. Flaherty KR, Wells AU, Cottin V, et al. Nintedanib in progressive fibrosing interstitial lung diseases. N Engl J Med. 2019;381:1718-1727. doi:10.1056/NEJMoa1908681.
44. Behr J, Prasse A, Kreuter M, et al. Pirfenidone in patients with progressive fibrotic interstitial lung diseases other than idiopathic pulmonary fibrosis (RELIEF): a double-blind, randomised, placebo-controlled, phase 2b trial. Lancet Respir Med. 2021;9:476-486. doi:10.1016/S2213-2600(20)30554-3.
45. Distler O, Highland KB, Gahlemann M, et al. Nintedanib for systemic sclerosis-associated interstitial lung disease. N Engl J Med. 2019;380:2518-2528. doi:10.1056/NEJMoa1903076.
46. Guyatt GH, Oxman AD, Vist GE, Kunz R, Falck-Ytter Y, Alonso-Coello P, Schunemann HJ; GRADE Working Group. GRADE: an emerging consensus on rating quality of evidence and strength of recommendations. BMJ. 2008;336:924-926. doi:10.1136/bmj.39489.470347.AD.
47. Richeldi L, Azuma A, Cottin V, et al. Nerandomilast in patients with idiopathic pulmonary fibrosis. N Engl J Med. 2025;392:2193-2202. doi:10.1056/NEJMoa2414108.
